# Metastatic suppression by DOC2B is mediated by inhibition of epithelial-mesenchymal transition and induction of senescence

**DOI:** 10.1007/s10565-021-09598-w

**Published:** 2021-03-24

**Authors:** Samatha Bhat, Divya Adiga, Vaibhav Shukla, Kanive Parashiva Guruprasad, Shama Prasada Kabekkodu, Kapaettu Satyamoorthy

**Affiliations:** 1 Department of Cell and Molecular Biology, Manipal School of Life Sciences, Manipal Academy of Higher Education, Karnataka, 576104 India; 2 Department of Ageing Research, Manipal School of Life Sciences, Manipal Academy of Higher Education, Karnataka, 576104 India

**Keywords:** DOC2B, Epithelial-mesenchymal transition, Metastasis, Senescence, Calcium, Cervical cancer, Abbreviations, BAPTA-AM-1,2-bis(o-aminophenoxy) ethane-N, N, N′, N′- tetraacetic acid ester, SA-βga-lSenescence-associated β galactosidase, SASP-Senescence-associated secretory phenotype, EMT-Epithelial to mesenchymal transition

## Abstract

Senescence induction and epithelial-mesenchymal transition (EMT) events are the opposite sides of the spectrum of cancer phenotypes. The key molecules involved in these processes may get influenced or altered by genetic and epigenetic changes during tumor progression. Double C2-like domain beta (DOC2B), an intracellular vesicle trafficking protein of the double C2 protein family, plays a critical role in exocytosis, neurotransmitter release, and intracellular vesicle trafficking. DOC2B is repressed by DNA promoter hypermethylation and functions as a tumor growth regulator in cervical cancer. To date, the molecular mechanisms of DOC2B in cervical cancer progression and metastasis is elusive. Herein, the biological functions and molecular mechanisms regulated by DOC2B and its impact on senescence and EMT are described. DOC2B inhibition promotes proliferation, growth, and migration by relieving G0/G1-S arrest, actin remodeling, and anoikis resistance in Cal27 cells. It enhanced tumor growth and liver metastasis in nude mice with the concomitant increase in metastasis-associated CD55 and CD61 expression. Inhibition of EMT and promotion of senescence by DOC2B is a calcium-dependent process and accompanied by calcium-mediated interaction between DOC2B and CDH1. In addition, we have identified several EMT and senescence regulators as targets of DOC2B. We show that DOC2B may act as a metastatic suppressor by inhibiting EMT through induction of senescence via DOC2B-calcium-EMT-senescence axis.

**Graphical abstract:**

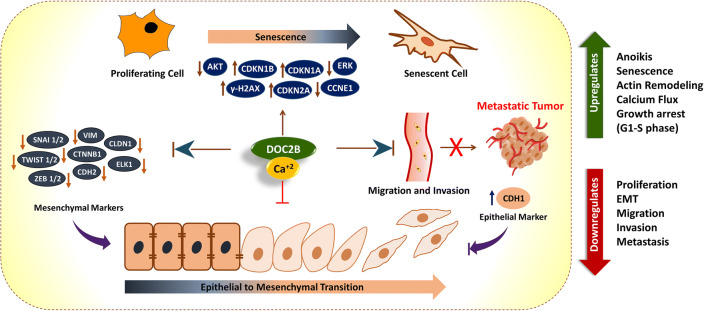

**Supplementary Information:**

The online version contains supplementary material available at 10.1007/s10565-021-09598-w.

## Introduction

As a key protein involved in intracellular vesicle trafficking, double C2-like domain beta (DOC2B), a member of the double C2 protein family located in Chr.17p13.3, regulates numerous physiological processes including exocytosis, neurotransmitter release, and intracellular vesicle trafficking (Kojima et al. 1996; Sakaguchi et al. 1995). DOC2B interacts with SNARE complex (SNAP25, STX1A, and VAMP2), STX4, and STXBP3 and competes with SYT1 (Aslamy and Thurmond 2017). DOC2B enhances immune-availability of syntaxin-1 and inhibits Ca^2+^ channels (Toft-Bertelsen et al. 2016). Among the two C2 domains, C2B is the primary calcium sensor while, C2A enhances plasma membrane association of C2B (Giladi et al. 2013). Translocation of DOC2B to the plasma membrane is calcium-dependent and requires phosphatidylinositol (4, 5)-bisphosphate (Michaeli et al. 2017). Recently, vesicle priming function and the role of DOC2B in type 1 diabetes were also reported (Houy et al. 2017). Studies have revealed the protective role of DOC2B against inflammatory damage in β cells (Aslamy et al. 2018).

The epithelial-mesenchymal transition (EMT) is a multi-step ontogenesis process driven by growth factors, cytokines, and extracellular matrix proteins resulting in the transformation of polar and non-motile epithelial cells into loosely organized and motile mesenchymal cells (Dongre and Weinberg 2019). EMT is a normal cellular process that takes place during embryogenesis and organ development. This transition involves loss of epithelial markers such as E-cadherin (CDH1), claudins (CLDNs), occludins (OCLN), plakophilins (PKP), cytokeratins (CK), and desmoplakin (DSP), gain of mesenchymal markers such as vimentin (VIM) and N-cadherin (CDH2) and EMT-related transcription factors (TFs) namely Twist family BHLH transcription factors 1 and 2 (TWIST1, TWIST2), Snail family transcriptional repressors 1 and 2 (SNAI1, SNAI2), and Zinc finger E-box binding homeoboxes 1 and 2 (ZEB1, ZEB2) (Kalluri and Weinberg 2009). Cytokine and growth factor signaling, particularly transforming growth factor beta (TGF-β), vascular endothelial growth factor (VEGF), fibroblast growth factor (FGF), epidermal growth factor (EGF), Wnt, Notch, interleukin 6 (IL6), hypoxia, hedgehog, and bone morphogenetic protein (BMP), can induce EMT (Gonzalez and Medici 2014; Witsch et al. 2010). Aberrant activation of EMT has attracted considerable attention as a possible reason for tumor evolution, metastasis, and therapeutic resistance (Adiga et al. 2021; Adiga et al. 2020; Thiery 2002). Abnormal expression of EMT-TFs and EMT-signalling pathways play a critical role in enhanced invasiveness and metastasis in cervical cancer. Studies utilizing in vitro, in vivo, and cervical clinical specimens have convincingly identified and supported the role of EMT in cervical cancer metastasis (Lee et al. 2008). Thus, understanding the molecular events responsible for EMT might benefit overall survival and improve targeted intervention in cancer. In contrast to EMT, cellular senescence shows an opposing role by participating in tumor suppressive mechanisms. Studies have reported the cross-talk between senescence and EMT. For instance, cancer cells may overcome senescence by upregulation of EMT-TFs, notably TWIST1, ZEB1, and ZEB2 to induce EMT phenotype and promote metastasis (Smit and Peeper 2010). Intracellular Ca^2+^ can also regulate both senescence and EMT (Martin and Bernard 2018; Stewart et al. 2015). Thus, inhibiting EMT signaling might be more beneficial as it can induce senescence, apoptosis, and other modes of cell death, thereby preventing invasion and metastasis.

We have previously reported that DOC2B is expressed in normal cervical cells, and its expression is substantially reduced in cervical cancer and cervical cancer cell lines such as SiHa, HeLa, and CaSki by promoter hypermethylation. Retroviral-mediated ectopic expression of DOC2B significantly inhibited growth rate, proliferative potential, and migratory and invasive properties of SiHa cells by inducing cell cycle arrest. These findings show that DOC2B is a tumor growth regulator in cervical cancer (Kabekkodu et al. 2014)*.* In the present study, we show that DOC2B significantly inhibits cancer cell metastasis in vivo. Mechanistically, DOC2B physically interacts with CDH1 and SNAI1 leading to the downregulation of EMT signaling with concomitant induction of the senescence pathway. Separately, we show that DOC2B induced senescence, and inhibition of EMT requires Ca^2+^.

Our results demonstrate the novel mechanism of DOC2B-mediated EMT and senescence regulation which can eventually modulate metastasis in cervical cancer. We propose that targeting DOC2B-calcium-EMT-senescence axis could be potential strategy to develop an effective treatment for metastasis in cervical cancer.

## Methods

### Cell culture

SiHa and Cal27 cell lines were procured from the ATCC (Manassas, VA, USA). Foreskin fibroblast cell line, generated at Manipal School of Life Sciences, MAHE, Manipal, was used in the study. The cell lines were cultured in complete media (DMEM + 10% FBS). The cell culture media and FBS were purchased from Himedia, India. All the cell lines used in the study were checked for cross contamination using microsatellite markers. For calcium depletion experiments, cells were pre-treated with 10 μM BAPTA-AM (Sigma-Aldrich, USA)) for 1 h and subsequently used for all experiments.

### shRNA-mediated *DOC2B* knockdown in Cal27 cells

SiHa and Cal27 cells were chosen for overexpression and knockdown experiments, respectively, as these cells show differences in baseline DOC2B expression. While DOC2B expression is downregulated SiHa, its expression level is higher in Cal27 cells. The retroviral transduction and development of SiHa cells expressing *DOC2B* were described earlier (Kabekkodu et al. 2014). The knockdown of *DOC2B* in Cal27 cells was performed using lentiviral shRNA against human *DOC2B,* cloned in piLenti-siRNA-GFP plasmids (Applied Biological Material, USA). *DOC2B*-knockdown cells were selected using 2 μg/mL of puromycin (Sigma-Aldrich, USA ) and the knockdown efficiency was tested using RT-PCR and western blot (Kabekkodu et al. 2014).

### Localization of DOC2B

The complete cDNA encoding *DOC2B* was isolated from pCMV-Entry-DOC2B (Origin, USA) and subcloned into the pEGFPC-1 vector to generate pEGFPC1-DOC2B. pEGFPC-1 and pEGFPC1-DOC2B were transfected into SiHa cells, and stable clones were isolated under 400 μg/ml of G418 selection (Sigma-Aldrich, USA) for 21 days. The localization of DOC2B was examined using a laser-scanning microscope SP-8 (Leica Microsystems, Germany) with a × 100 objective.

### Anchorage-dependent colony formation assay

Approximately, 500 cells were seeded in a 6-cm cell culture plate. After 14 days, the culture medium was removed and washed thrice with PBS. Following this, cells were incubated with a staining solution (0.5% crystal violet in methanol) for 10–15 minutes. Excess stain was discarded and washed with PBS, and stained colonies were counted using a microscope as published previously (Bhat et al. 2021; Hu et al. 2013).

### Anchorage-independent colony formation assay

1 × 10^3^ cells/well in 0.3% Nobel agar (Sigma-Aldrich, USA) prepared in DMEM with 10% FBS was overlaid above 3 mL of 0.6% bottom agar containing DMEM+10%FBS. After 4 weeks of culturing in complete media, colonies were counted using a microscope (Bhat et al. 2021; Kaneda et al. 2004).

### Cell doubling and growth curve analysis

Cells (2 × 10^4^) were cultured in a 35-mm cell culture dishes for 5 days to analyze the growth curve. Cells were harvested by trypsin at indicated time points, and cell counting was carried out using a hemocytometer. The cell doubling time was calculated using http://www.doubling-time.com/compute.php.

### Anoikis assay

1 × 10^5^ cells were cultured in a poly-HEMA (Sigma-Aldrich, USA)-coated plate. The rate of anoikis was determined by staining the cells with propidium iodide (10 μg/mL in PBS, Sigma-Aldrich, USA) and analysed using FACS (BD Biosciences, USA) (Haraguchi et al. 2008).

### Senescence assay

Cells were grown on 35-mm dishes, serum starved, fixed with 4% paraformaldehyde (Sigma-Aldrich, USA), incubated with staining solution containing 5 mM K3Fe(CN)6, 5 mM K4Fe(CN)6, 30 mM sodium phosphate buffer, 150 mM NaCl, 2 mM MgCl2, and 1 mg/ml X-Gal at pH 6.0 at 37 °C for 12–16 h. All the chemicals were purchased from Sigma-Aldrich, USA. To analyze the induction of senescence in tumor xenografts, tissue cryo-sections were fixed with 4% paraformaldehyde for 5 min and then incubated with staining solution (5 mM K3Fe (CN)6, 5 mM K4Fe(CN)6, 0.1 M citrate buffer, 150 mM NaCl, 2 mM MgCl2, and 1 mg/ml X-Gal at pH 4.0) at 37 ^°^C for 4 h. All the chemicals used were procured from Sigma-Aldrich, USA. Excess stain was removed; cells were washed with PBS, and images were captured using DP80 camera attached to BX51 microscope (Olympus, Japan). The cells positive for SA-β-gal staining were counted from five independent fields to calculate the percentage of positive cells (Wen et al. 2014).

### Cell cycle and apoptosis assay

Distribution of cells at various phases of cell cycle was evaluated by making use of BrdU flow Kit (BD Biosciences, USA). Cells were grown for 48 h in serum-free DMEM followed by addition of BrdU (10 μM/mL) for 30 min at 37 °C and cultured in complete medium for the indicated times. Cell cycle distribution was assessed by propidium iodide (Sigma-Aldrich, USA) staining (10 μg/mL in PBS) and analyzed using a flow cytometer with Cell Quest software (BD Biosciences, USA) (Haraguchi et al. 2008).

### Migration assay

In a 6-well plate, cells were cultured to 90% confluency. Following PBS wash, cells were cultured in the serum-free medium for 24 h. A scratch was made at the center of the plate using a 200-μL micro-tip. Subsequently, the cells were cultured in the presence of complete medium and monitored at the indicated time using a progress camera (Jenoptik AG, Germany) attached to CKX41 Microscope (Olympus, Japan). The rate of cell migration and migration index were estimated as per published protocols (Xu et al. 2012).

### Three-dimensional invasion assay

Cells were grown in a chambered glass slide (Ibidi, Germany) and treated with 10 μM BAPTA-AM (Sigma-Aldrich, USA) for 1 h. Following this, 200 μl collagen I mix [collagen R-1.8 mg/ml (Serva, Germany), 5X DMEM (Himedia, India)—200 μl per ml of collagen I mix, NaOH (Alfa Aesar, USA)—8 ul per ml of collagen I mix] was added to each well and thermally gelled at 37 °C for 2–3 h. Collagen gel was layered with 100 μl of media with 20% FBS and incubated for 48 h. After incubation, media was aspirated, gels were washed with PBS, fixed with 3% paraformaldehyde (Sigma-Aldrich, USA) (15mins), permeabilized with 0.5% Triton X-100 (Alfa Aesar, USA) (30 min), and blocked with 1% BSA (Himedia, India) (30 min). Cells were then stained with Phalloidin-TRITC (Sigma-Aldrich, USA) (for 90 min) and Hoechst (Himedia, India) (for 15 min) at room temperature. Invaded cells were imaged using laser scanning confocal microscope with × 63 oil immersion objective (Leica Microsystems, Germany). Confocal Z slices were collected for each well at 40 μm from the bottom, and sequential Z slices were used to construct the 3D images (Yang and Yang 2013).

### In vivo tumorigenicity and metastasis assay

For tumorigenesis, 5–6-week old female athymic nude mice (5 per group) were used after obtaining approval from the MAHE animal ethics committee. Scrambled and *DOC2B* knockdown Cal27 cells (2.5 × 10^6^) were mixed with Matrigel (BD Biosciences, USA) (1:1 ratio) and transplanted subcutaneously into the animals (Xu et al. 2012). Growth of the tumor was monitored for over 2 months. *V* = ab^2^/2 formula was used to calculate the tumor volume, where in “V” is the tumor volume, “a” is the length and “b” is width of the tumor. For the in vivo metastasis assay, 2 × 10^6^ cells suspended in 0.15 ml PBS were injected through the tail vein of 5–6-week old nude mice (*n* = 5/group). On the 6th week, animals were sacrificed, organs were excised, and paraffin blocks were prepared.

### Hematoxylin-Eosin (H&E) and Masson’s trichrome staining

Tissues from each animal were formalin fixed, and paraffin blocks were prepared according to standard protocol. Tumor tissue cryosections (5 μM) were stained with H&E and Masson’s trichrome stains (Sigma-Aldrich, USA). The slides were evaluated by expert pathologists.

### Cell surface marker analysis

The transfected cells (1 × 10^6^) were detached using EDTA (10 mM in PBS, Thermo Fisher Scientific, USA), washed with PBS, and incubated at room temperature with anti-CD55 and CD61 (2 μg) antibodies in the dark (BD, USA) for 30 min and washed with PBS. Following this, the stained cells were resuspended in PBS. The CD55 and CD61 expression were examined using a FACS Calibur (BD, USA) and Cell Quest software.

### Gene expression microarray

Total RNA was isolated using TRIzol reagent (Invitrogen, USA), amplified and labelled using Low RNA Input Linear Amplification Kit (Agilent, USA). In brief, 1.65 μg of labeled RNA was hybridized onto a 4X44K Human expression array and scanned using a 2565 BA scanner (Agilent Technologies, USA). Feature extraction software 10.7 (Agilent Technologies, USA) and Gene Spring Software 11.0 (Agilent Technologies, USA) were used for the extraction of gene expression microarray data. The genes with > twofold or < two fold were considered as differentially expressed. The gene ontology, pathways, and gene to gene interaction were performed using KEGG (www.genome.jp/kegg/pathway), DAVID (https://david.ncifcrf.gov/summary.jsp) and Gene Spring software. The pathway enrichment analysis was performed using Enricher (http://amp.pharm.mssm.edu/Enrichr/).

### EMT induction and morphological assessment

Transfected cells (1 × 10^5^) were grown on a coverslip in serum-free medium and treated with EGF-1 (50 ng/mL) (Sigma-Aldrich, USA), FGF-2 (5 ng/mL) (Sigma-Aldrich, USA), TGF-β (2 ng/mL) (Himedia, India), IL-6 (25 ng/mL) (Sigma-Aldrich, USA), and TNF-α (10 ng/mL) (Sigma-Aldrich, USA) for 72 h and stained with phalloidin-TRITC as described earlier (Hu et al. 2011). The images were captured using SP-8 confocal microscope using a × 100 objective.

### Semi quantitative reverse transcriptase PCR

TRIzol reagent (Life Technologies, USA) was used to extract RNA from 48 h-serum-starved transfected cells. High-capacity cDNA archive kit was used to synthesize cDNA (Life Technologies, USA). The primers and conditions used for PCR are provided as Table 1. Using NIH ImageJ software (http://imagej.nih.gov/ij/), the relative gene expression levels were quantified densitometrically with β-actin as an internal control.

**Table 1:** The list of primers used

Primer name	Primer sequence (5′–3′)	Annealing temperature	Product size
Reverse transcriptase PCR (RT-PCR)			
DOC2B-RT-PCR-F	TGGTGTGGTTCTGGGCATCCACG	60 °C	103 bp
DOC2B-RT-PCR-R	TGGGAGCTCGCTGGTGAGCGTG		
ACTB –RT-PCR-F	GACGACATGGAGAAAATCTG	60 °C	132 bp
ACTB- RT-PCR-R	ATGATCTGGGTCATCTTCTC		
VIM-RT-PCR-F	ATCCAAGTTTGCTGACCTCTCTGAG	60 °C	102 bp
VIM-RT-PCR-R	AGGGACTGCACCTGTCTCCGGT		
CTNNB1-RT-PCR-F	GATATTGGTGCCCAGGGA	60 °C	127 bp
CTNNB1-RT-PCR-R	CACCCATCTCATGTTCCATC		
TW1-RT-PCR-F	GGCTCAGCTACGCCTTCTC	60 °C	130 bp
TW1-RT-PCR-R	TCCTTCTCTGGAAACAATGACA		
TW2-RT-PCR-F	GCAAGAAGTCGAGCGAAGAT	57.5 °C	92 bp
TW2-RT-PCR-R	GCTCTGCAGCTCCTCGAA		
SNAI1-RT-PCR-F	TATGCTGCCTTCCCAGGCTTG	60 °C	143 bp
SNAI1-RT-PCR-R	ATGTGCATCTTGAGGGCACCC		
SNAI2-RT-PCR-F	ATCTGCGGCAAGGCGTTTTCCA	60 °C	127 bp
SNAI2-RT-PCR-R	GAGCCCTCAGATTTGACCTGTC		
ZEB1-RT-PCR-F	TCCTGAGGCACCTGAAGAGG	57.5 °C	139 bp
ZEB1-RT-PCR-R	CAGAGAGGTAAAGCGTTTATAGCC		
CDH1-RT-PCR-F	GCCTCCTGAAAAGAGAGTGGAAG	60 °C	131 bp
CDH1-RT-PCR-R	TGGCAGTGTCTCTCCAAATCCG		
CDH2-RT-PCR-F	CCTCCAGAGTTTACTGCCATGAC	60 °C	149 bp
CDH2-RT-PCR-R	GTAGGATCTCCGCCACTGATTC		

### Immunoblotting

Whole-cell protein (20–50 μg) was initially separated using 8% SDS-PAGE and subsequently, transferred onto Nitran membrane (Sigma-Aldrich, USA). Next, the membranes were blocked with BSA (5%), and incubated separately with anti-DOC2B (1:5000; Proteintech, USA), anti-CTNNB1 and CDH1 (1:3000) (Developmental Studies Hybridoma Bank, University of Iowa, USA), p-ELK-1(Ser383), total ELK-1, p-AKT (Ser473), total AKT, p-ERK1/2(Thr202/Tyr204), total- ERK1/2 (Thr202/Tyr204), p-p38MAPK (Thr180/Tyr 182), total- p38MAPK, MacroH2A1.2, Tri methyl H3 lys9, SNAI1, SNAI2, ZEB1 and β-actin (1:3000, Cell Signaling Technologies, USA), CCNE (HE12), GSK3α/β, CDKN2A, CDKN1A, CDKN1B (1:3000, Santa Cruz Technologies, USA) TWIST2 (1:2000, Abcam, Cambridge, USA) and CLDN1, CDH2 VIM (1:3000, Cloud Clone, USA) at 4 °C, and then with anti-mouse IgG-HRP or anti-rabbit IgG-HRP (1:5000) (Cell Signaling, USA) secondary antibodies. SuperSignal™ West Pico Chemiluminescent Substrate (Thermo Scientific, USA) was used for visualization of proteins in the membranes using Image Quant LAS 4000 (GE Healthcare, USA).

### Small G proteins pull down assay

The RAS, RAC1, and CDC42 activation were evaluated by pull down assay kit (Millipore, USA) as per the manufacturer’s protocol. For RAS activation, DMEM containing 10% FBS and for RAC1/CDC42 activation, DMEM containing 10% FBS and 100 ng/ml PMA were used for 5 min. The levels of active forms of RAS, RAC1, and CDC42 in pulldown proteins were tested using mouse anti-RAS, mouse anti-RAC1, or mouse anti-CDC42 antibodies (Millipore, USA) by western blotting.

### TCF/LEF transcriptional activity

In a 12-well plate, 1 × 10^5^ of stably transfected cells were co-transfected with 0.5 μg/well of either TOPFLASH or FOPFLASH reporter plasmids (Upstate Biotechnology, USA) along with 25 ng/well pRL-SV40 using Lipofectamine LTX (Bernard et al. 2008). The luciferase assay readings were normalized against the pRL-TK vector. The TCF/LEF transcriptional activity was estimated by taking the ratio between pTOPFLASH vs. pFOPFLASH luciferase activity.

To assess the effect of DOC2B on wild type and mutant CTNNB1 (S33Y) inducible TCF/LEF activity, stable cell lines were co-transfected with 100 ng/well of wild type and mutant CTNNB1, 500 ng of pTOP- or pFOP- FLASH, and 25 ng of pRL-SV40. The Dual Luciferase™ Reporter assay kit (Promega, USA) was used to measure the TCF/LEF reporter activity 48-h post transfection as published previously (Kuroda et al. 2006).

### Immunofluorescence and confocal microscopy

Cells were cultured on sterile coverslip, fixed with 4% paraformaldehyde (10 min), permeabilized with 0.5% Triton X-100 (Alfa Aesar, Germany), and blocked with 0.05% Tween-20 (Sigma-Aldrich, USA) prepared in 3% BSA in PBS for 1 h and incubated overnight with phospho-H2AX, MacroH2A1.2 and Tri methyl H3 lys9 (1:100; Cell Signaling Technologies, USA), anti-CTNNB1 (1:100; Transduction Laboratories, USA), DOC2B (1:200, Protein Tech, USA), and CDH1 (1:1500; Developmental Studies Hybridoma Bank, University of Iowa) antibodies and then with secondary antibody wither labelled with FITC or TRITC (Thermofisher, USA). The cells were counterstained with Hoechst (Himedia, India) and mounted using Vectashield (Vector Laboratories, USA) onto a microscopic slide. Leica TCS SP8 confocal platform equipped with DMi8 microscope (Leica Microsystems, Germany) was used for image acquisition using × 100 oil immersion objectives. For senescent foci analysis, at least 100 cells were counted and the nuclei displaying ≥ 10 discrete dots of brightness were counted as senescence-positive cells.

### Co-immuno-precipitation assay

For co-immuno-precipitation assay, cell lysates were prepared from *DOC2B*-expressing SiHa cells using NP40 lysis buffer. Cell lysates (250 μg) were incubated with 1 μg of Rabbit IgG and anti-DOC2B antibodies for overnight at 4 °C. Subsequent incubation was performed with protein A/G immuno-magnetic beads (Sino Biologicals, China) for 6 h at 4 °C. Furthermore, the beads were washed with 0.01% PBST; the immuno-precipitated complexes were collected from the beads as per the manufacturer’s guidelines (Sino Biologicals, China) and then subjected to SDS-PAGE and immuno-blotting.

### Statistical analysis

The Student’s *t* test (2-tailed unpaired) was performed using GraphPad prism (Free online tool). Data represented as mean ± SD with *P* < 0.05 was considered as statistically significant. The experiments were conducted in duplicated and repeated 3 times.

## Results

### DOC2B is localized to the plasma membrane

**Fig. 1:**
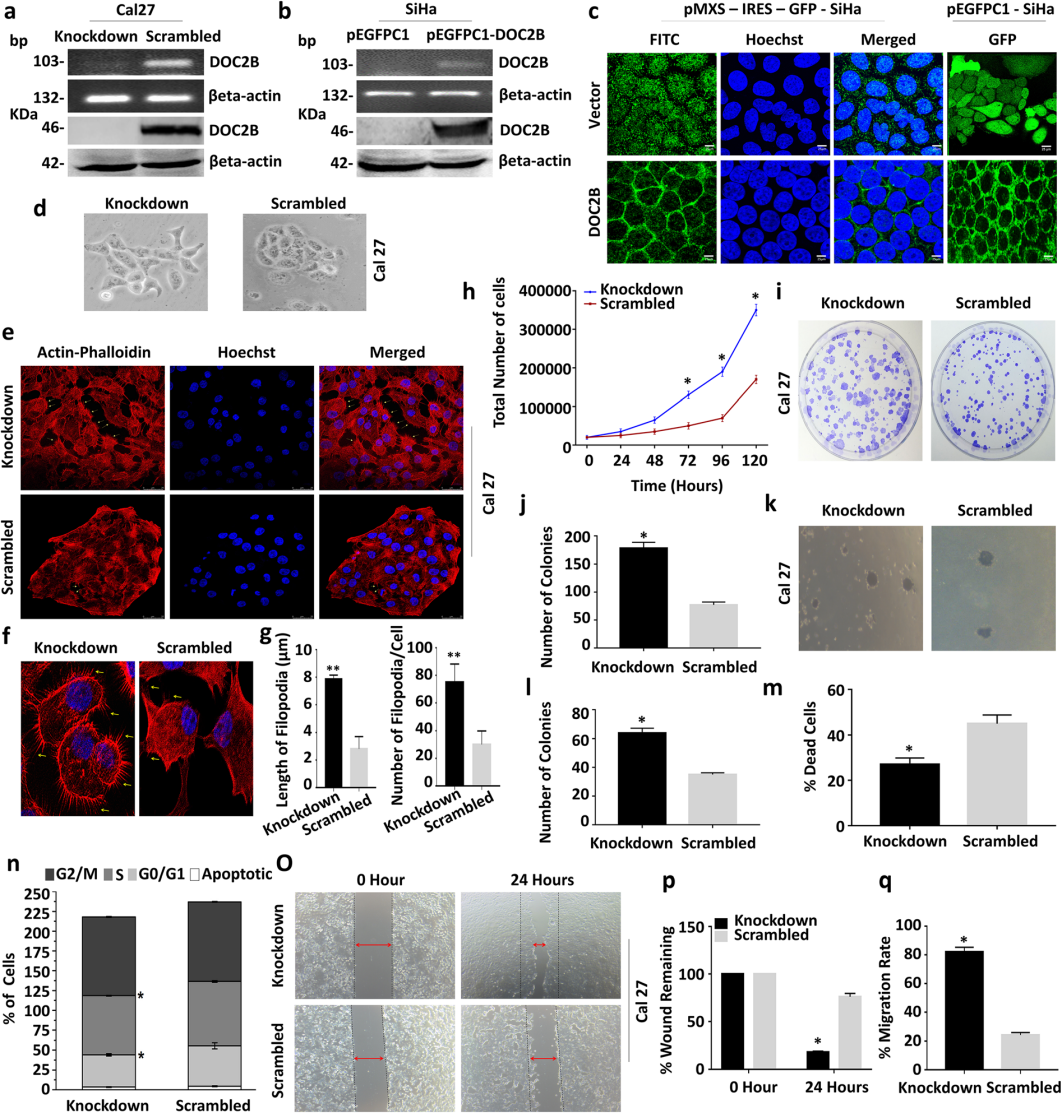
Effect of *DOC2B* knock down in Cal27 cells. **a** Representative images confirming the knockdown of DOC2B expression (upper panel) and protein (lower panel). Knockdown and scrambled represents the Cal27 cells transfected with shRNA against *DOC2B* and negative control shRNA respectively. **b** Representative RT-PCR (upper panel) and western blot (lower panel) images confirming the expression of EGFP-DOC2B in SiHa cell. **c** Images showing the localization of DOC2B predominantly in the plasma membrane in SiHa cells. **d** Knockdown of *DOC2B* in Cal27 cells induced distinct morphological changes. **e** *DOC2B* knockdown in Cal27 cells induced rearrangement of actin fibers, increased the length and number of filopodia and lamellipodia. **f** Representative confocal images showing increased number of filopodia in DOC2B knockdown Cal27 cells **g** Bar graph representing the length and number of filopodia in DOC2B knockdown and Scrambled cells. **h** The knockdown of *DOC2B* significantly increased the cell proliferation by decreasing the cell doubling time (24.7 h) as opposed to scrambled control cells (34.5 h, *P* < 0.005). **i**, **j**. The knockdown of *DOC2B* increased anchorage dependent tumor growth. The size and number of colonies were significantly increased upon knockdown (*P* < 0.05). **k**, **l** Represent the anchorage independent colony growth and the quantitative estimations. The knockdown of *DOC2B* enhanced the number and increased the size of the colony when compared with scrambled cells (*P* < 0.05). **m** The anoikis-mediated cell death was significantly lower in *DOC2B* knockdown Cal27 cells as opposed to scrambled cells (24.45 ± 3.18 vs. 46.7 ± 0.98, *P* = 0.01). **n** The cell cycle analysis revealed that knocking down of *DOC2B* in Cal27 cells relived the G1/S phase block. **o** Representative image of cell migration assay. Knockdown of *DOC2B* increases cell migration which is evident by faster closure of wound in comparison with scrambled cells. **p**–**q** Representative graph showing the quantitative analysis of migration. Quantitative analysis at 24 h showed increase in wound closure rate (81.6% vs. 26.8%) between *DOC2B* knockdown and scrambled cells with complete wound closure at the end of 24 h. *P < 0.05 by independent Student’s *t* test was considered as statistically significant

To investigate the function of DOC2B, we generated (i) retroviral-mediated *DOC2B* overexpression in SiHa cells, (ii) lentiviral-mediated knockdown of *DOC2B* in Cal27 cells and (iii) pEGFPC-1-based *DOC2B* overexpression in SiHa cells for live imaging and localization studies. Knockdown was by a lentiviral approach using shRNA against DOC2B mRNA. There was more than a 95% decrease in mRNA and protein expression (Fig. 1a). We have cloned the cDNA encoding DOC2B into a pEGFPC-1 vector and transfected into SiHa cells for localization studies (Fig. 1b). Subsequently, immunofluorescent microscopy and live imaging were performed using pEGFPC1-DOC2B-expressing SiHa cells to show that DOC2B is localized to the plasma membrane (Fig. 1c).

### Knocking down of *DOC2B* induces morphological changes characteristic of metastatic cells

*DOC2B* knockdown cells showed distinct morphological changes, altered actin rearrangements, reduced cell to cell adhesion, and increased number and length of filopodia when compared to scrambled construct transfected control cells (Fig. 1d, e, f, g).

### *DOC2B* influences growth and proliferation in vitro

We observed that knockdown of *DOC2B* in Cal27 cells increased anchorage-dependent and independent colony growth (size and number) and proliferation by reducing cell doubling time as opposed to scrambled control cells (Fig. 1h–l). The doubling time of the cells were 49.69 h for scrambled cells as opposed to 33.47 h for DOC2B knockdown cells.

### *DOC2B* knockdown inhibits anoikis-mediated-cell death and relieves G0/G1-S arrest and induces Cal27 cell migration

We investigated the association between DOC2B manipulation and apoptosis. There was no significant change in apoptosis rate in an anchorage-dependent condition. However, knockdown of *DOC2B* significantly inhibited anoikis-mediated cell death when compared to scrambled cells (Fig.1m). Knockdown resulted in decrease in a G0/G1 and increase in S phase cells (Fig. 1n), respectively. Quantitative analysis at 24 h showed an increase in wound closure and migration rate in *DOC2B* knockdown cells than the scrambled cells (Fig. 1o–q). The findings were further confirmed by knocking down of DOC2B in DOC2B-SiHa cells by using lentiviral transduction (Supplementary Fig. 1)

### *DOC2B* affects tumor growth and metastasis in vivo

**Fig. 2:**
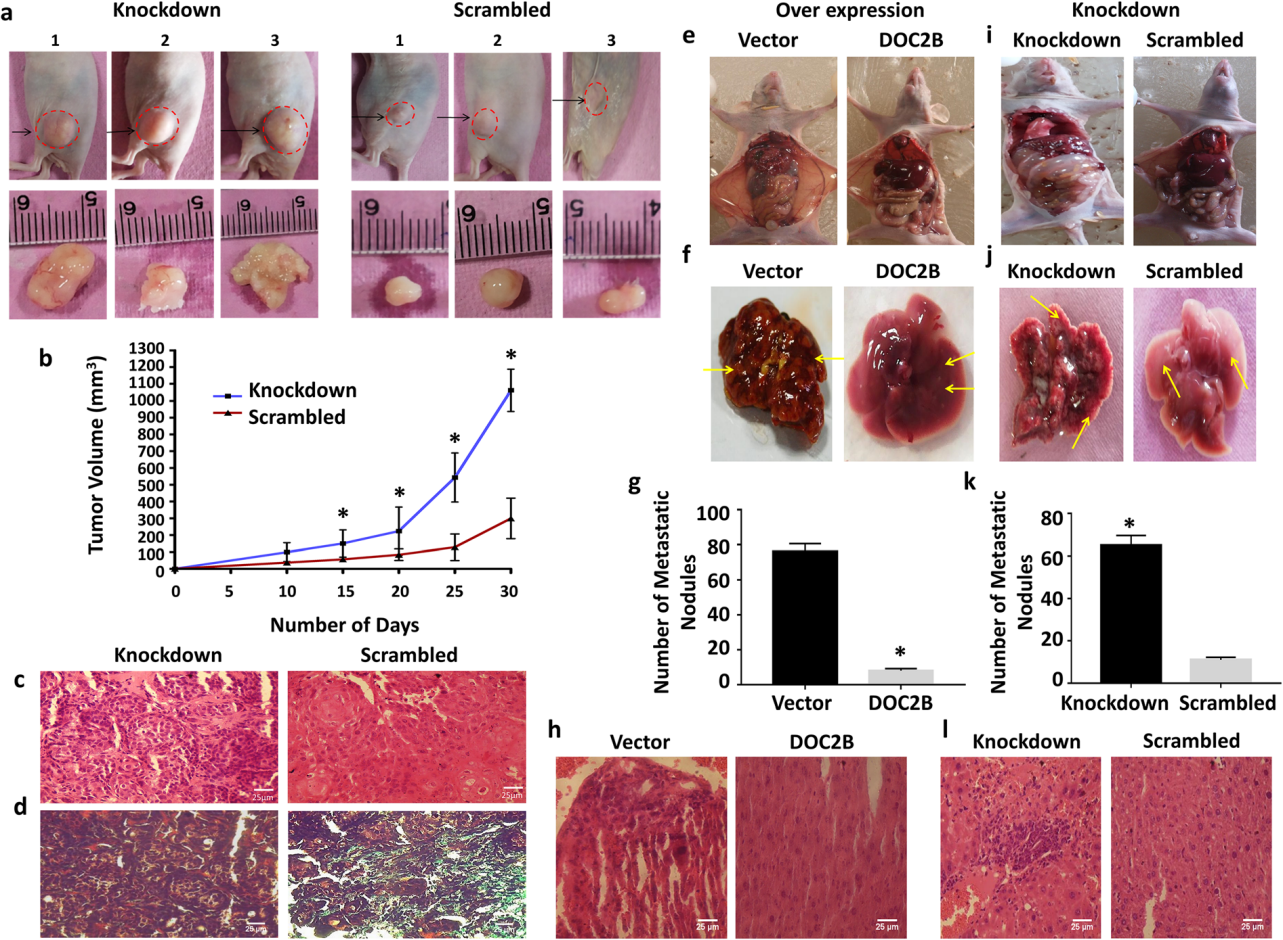
In vivo tumorigenicity and metastasis assay. **a** Silencing of *DOC2B* in Cal27 cells enhanced tumor growth in vivo. The nude mice (*n* = 3) were injected subcutaneously with 2 × 10^6^ of either *DOC2B* knockdown or scrambled cells and progressive tumor growth pattern were analyzed. **b** Tumor growth curves of *DOC2B* knockdown cells were compared with scrambled cells. The mice receiving knockdown cells formed progressively growing tumor when compared with mice receiving scrambled cells. Moreover, the tumor size/volume was significantly larger in group of mice receiving *DOC2B* silenced cells. The asterisk indicates statistical significance (*P < 0.05). **c** Representative image of the H&E staining of the tumor xenografts derived from *DOC2B* silencing experiments. Further, *DOC2B*-negative cells were predominantly/increasingly pleomorphic, spindle morphology cells showing prominent nuclei with altered nucleus to cytoplasmic ratio and abnormal mitosis. **d** Representative image of Masson’s trichrome staining of tumor xenografts with and without *DOC2B* knockdown. The presence of *DOC2B* significantly inhibited degradation or enhanced collagen synthesis as evident by Masson’s trichrome staining*.* **e**, **i** Representative image of mice showing the metastasized liver. The presence of *DOC2B* inhibited liver metastasis in vivo both in overexpression and knockdown models. **f**, **j** Representative images of metastasized liver in the presence and absence of *DOC2B* expression. **g**, **k** Quantitative analysis of number of metastatic nodules in *DOC2B*-positive and -negative cells in both overexpression (**g**) and knockdown experiments (**k**) respectively. **h**, **l** Representative H&E staining of liver sections (magnification, × 40). The mice receiving *DOC2B*-negative cells exhibited extensive liver metastases when compared to mice injected with *DOC2B*-positive cells in both overexpression and knockdown systems. The asterisk indicates statistical significance (*P < 0.05)

*DOC2B* knockdown Cal27 cells formed progressively growing tumors with significantly bigger tumor size and volume when compared with scrambled control cells (Fig. 2a, b). The histopathological examination of tumor cryosection showed that *DOC2B* knockdown increased the number of atypical cells, abnormal nucleus to cytoplasmic ratio, and the density of tumor cells with loosely aggregated cells (Fig. 2c). The decrease in collagen levels in *DOC2B*-negative cells was observed by Masson’s Trichrome staining (Fig. 2d). In vivo metastasis assay showed significantly reduced metastasis to the liver in mice receiving *DOC2B*-expressing cells when compared to mice receiving DOC2B-deficient cells (Fig. 2e, i). This was evident on the examination of microscopic metastatic nodules which are clearly visible, significantly more in number and bigger in size in the liver of mice receiving either control cells or knockdown cells (Fig. 2f, g, j, k). The H&E staining showed that mice receiving *DOC2B*-expressing cells displayed no or markedly decreased tumor cells in the liver (Fig. 2h, l).

### Identification of gene ontology, interactions, and pathways regulated by *DOC2B*

We have performed a gene expression microarray to identify the downstream pathways regulated by *DOC2B*. Microarray analysis identified 1353 genes (192 upregulated and 1161 downregulated) and 2304 genes (376 upregulated and 1928 downregulated) as differentially expressed upon overexpression and knockdown of *DOC2B* in SiHa and Cal27 respectively (Supplementary Fig. 2). We have identified 56 and 124 KEGG annotations related to cell proliferation, cell cycle, growth, migration, invasion, Wnt signaling, cell adhesion, MAPK, TGFβ, calcium, and EMT signaling as highly enriched (Supplementary Fig. 2  and 3 and Supplementary Tables 1 and 2). Overlapping analysis between the up- and downregulated genes in the overexpression and knockdown models identified 63 common genes (Supplementary Fig. 4 and Supplementary Table 3).

### DOC2B inhibits epithelial to mesenchymal transition

**Fig. 3:**
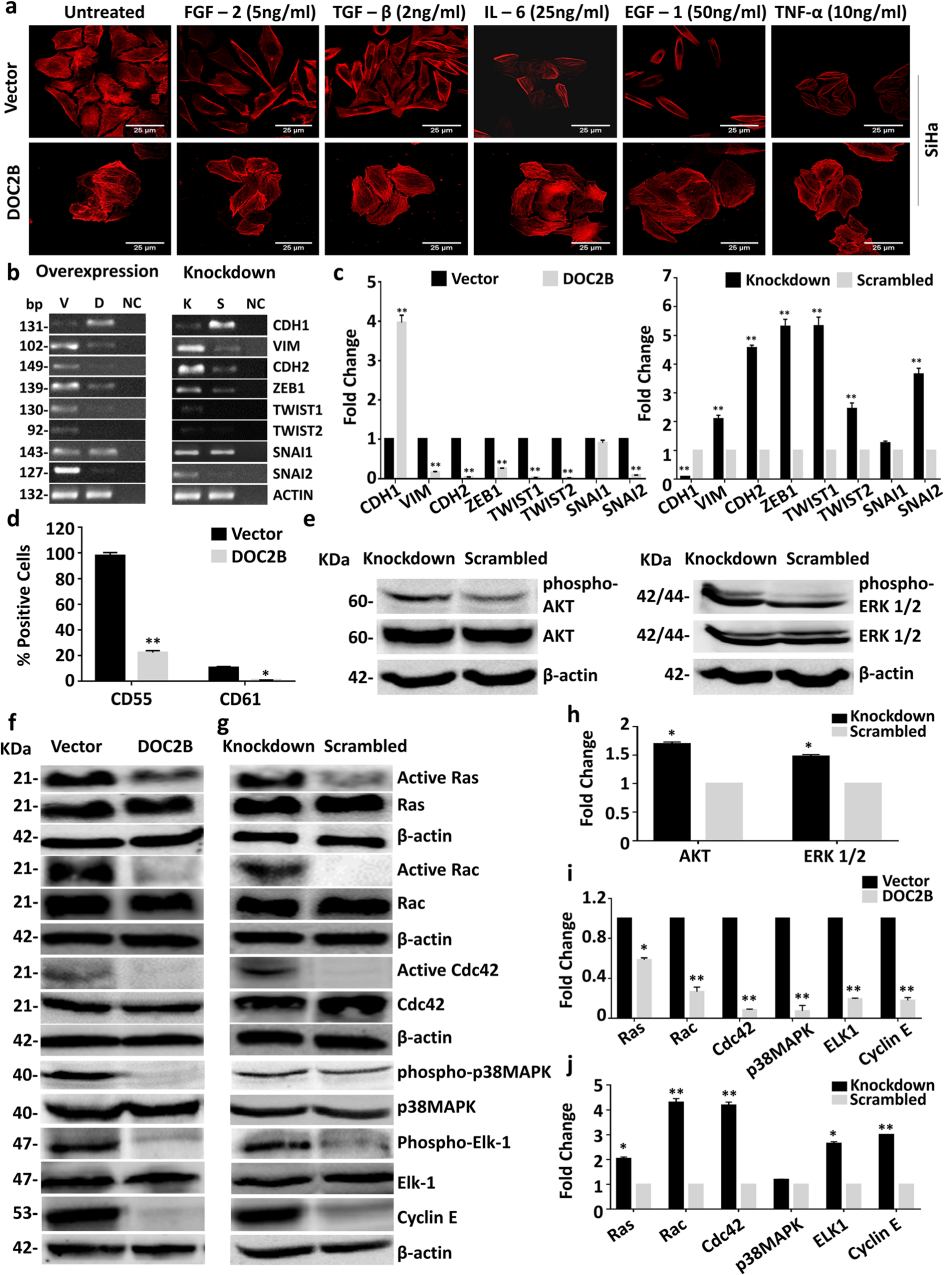
*DOC2B* acts as inhibitor of epithelial to mesenchymal transition (EMT). Control and *DOC2B*-expressing SiHa cells were treated with different EMT inducers for 72 h and analyzed for morphological changes. *DOC2B*-expressing cells did not show any significant morphological changes while control cells showed mesenchymal morphology upon treatment with EMT inducers. **a** Actin phalloidin staining upon treatment with EMT inducers showed that control cells displayed an elongated fibroblast-like morphology with scattered distribution, whereas *DOC2B*-overexpressing cells were more cobblestone-shaped with epithelial morphology. **b** RT-PCR analysis showing the downregulation of expression of *VIM*, *CDH2*, *TW1*, *TW2*, *SNAI1*, *SNAI2*, and *ZEB1* and upregulation of *CDH1* in the presence of *DOC2B* in both overexpression and knockdown cells along with their respective controls. V, D, and NC represents *DOC2B* over expression, control cells, and negative control; and K, S, and NC represents *DOC2B* knockdown, scrambled, and negative control respectively. **c** The bar graph representing the results of RT-PCR analyzed using Image J software. *P < 0.05 indicates statistical significance. **d** The bar diagram represents the cell surface markers tested by in vitro experiment using SiHa-DOC2B cells. Ectopic expression of *DOC2B* inhibited the expression of cell surface markers namely CD61, CD55 which are reported to confer stemness to variety of cell types. **e** Western blot showing the inhibition of AKT and ERK1/2 phosphorylation in *DOC2B* knockdown cells without altering the total protein. **f** Western blot showing the inhibition of active RAS, RAC1, CDC42, p38MAPK, ELK-1, and CCNE upon ectopic expression of *DOC2B* in SiHa cells as opposed to control cells. **g** The knockdown of *DOC2B* expression in Cal27 increased the active RAS, RAC1, CDC42, p38MAPK, ELK-1, and CCNE as opposed to scrambled cells. β-actin was used as the internal loading control in all the experiments. **h**, **i**, **j** Bar graph showing the quantitative analysis of expression of AKT, ERK1/2, RAS, RAC1 CDC42, p38 MAPK, ELK-1, and CCNE in DOC2B overexpression and knockdown cells

The gene expression microarray analysis identified key genes related to EMT signaling, targeted by *DOC2B* in both overexpression (up: 17 and down: 25 genes) and knockdown models (up: 57 and down: 62 genes) respectively (Supplementary Table 4). Treatment with EMT inducers did not induce any appreciable morphological change in *DOC2B*-expressing SiHa cells. Conversely, control cells readily converted into spindle-like mesenchymal type and showed an unorganized arrangement of actin (Fig. 3a). *DOC2B* inhibited EMT by enhancing the expression of epithelial gene (*CDH1)* with concomitant reduction in the expression of mesenchymal genes (*VIM*, *CDH2*) and EMT TFs (*TWIST1*, *TWIST2*, *SNAI1*, *SNAI2,* and *ZEB1)* when compared to respective *DOC2B*-deficient control cells (Fig. 3b, c). The expression of EMT markers in metastatic liver tissue in DOC2B overexpression model is shown in Supplementary Fig. 5.

### DOC2B inhibits key genes associated with proliferation and migration

The levels of metastatic markers, namely, CD55 and CD61, were significantly decreased in *DOC2B*-expressing cells upon comparison with control vector-transfected cells (Fig. 3d). The knockdown of *DOC2B* enhanced the phosphorylation of AKT1 (Ser473), ERK1/2 (Thr202/Tyr204), p38-MAPK, and ELK-1 without altering the expression of total protein in Cal27 cells (Fig. 3e–j). In both overexpression and knockdown cells, the presence of *DOC2B* significantly reduced the active form of RAS, RAC1, and CDC42 without altering the total protein levels (Fig. 3f, g, i, j). Taken together, our findings suggests that downregulation of the active forms of RAS, RAC1, and CDC42 may contribute to the anti-proliferative and anti-migratory functions of *DOC2B*. Besides, CCNE levels were also downregulated in cells expressing *DOC2B* when compared to control vector-transfected cells. However, the phosphorylation level of p38MAPK was significantly reduced only in the overexpression system.

### *DOC2B* expression induces senescence

**Fig. 4:**
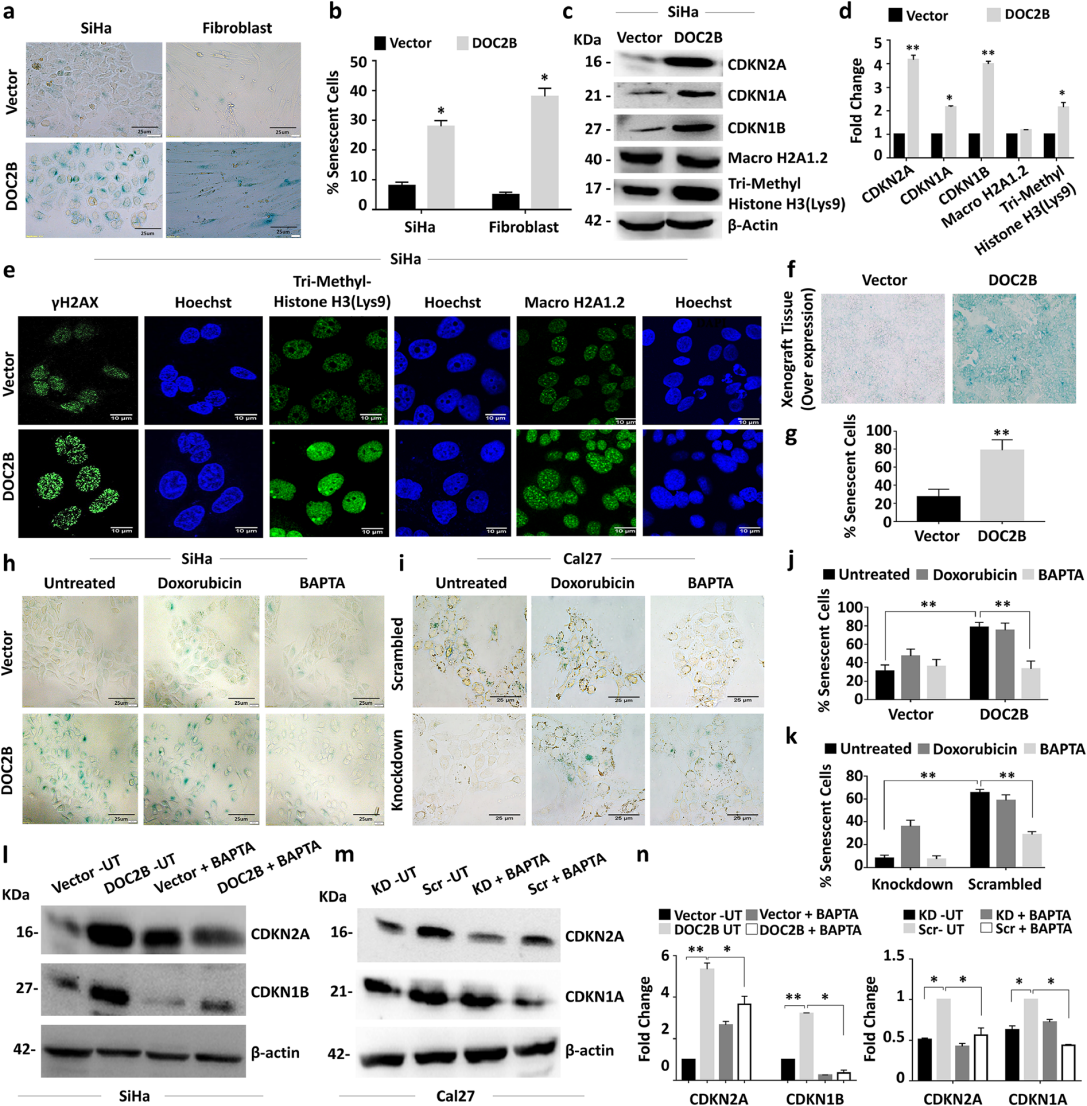
*DOC2B* induces senescence by induction of CDKN2A, CDKN1A, and CDKN1B and requires intracellular calcium. **a** Representative image showing the SA-β gal staining of control and DOC2B overexpressing SiHa and normal diploid fibroblast cells at × 40 magnification. The cells stained in blue color represent senescence-positive cells showing enlarged flattened morphology with cytoplasmic aggregates and were significantly more in *DOC2B* overexpressing SiHa and fibroblast cells as opposed to control SiHa and fibroblast cells. **b** The bar graph showing the quantitative analysis of SA-β-galactosidase-positive cells. The percentage of senescent cells were significantly higher in DOC2B-expressing cells as opposed to control cells (*P* < 0.05). **c** The western blot analysis for CDKN2A, CDKN1A, and CDKN1B, Tri-Methyl- Histone H3 (Lys9), and MacroH2A1.2. The level of CDKN2A, CDKN1A, and CDKN1B were significantly upregulated while Tri-Methyl-Histone H3 (Lys9) was slightly elevated upon DOC2B overexpression in SiHa cells. **d** Bar graph showing the expression levels of senescence associated proteins in DOC2B-expressing SiHa cells. **e** The γH2AX, Tri-Methyl-Histone H3 (Lys9), and MacroH2A1.2 immunostaining and analysis by confocal microscopy at × 100 magnification for senescent foci. The nuclear accumulation, number, and size of γH2AX and Tri-Methyl-Histone H3 (Lys9) foci were significantly increased, higher, and larger upon *DOC2B* overexpression in SiHa cells. The experiment was performed in duplicates and repeated 3 times, and the data was represented as mean ± SD for three independent experiments. **f** Representative images showing senescence induction in tumor xenograft tissue. Tumor sections from nude mice which received DOC2B-expressing cells showed higher senescence induction. **g** Bar graph indicating percentage senescent cells in tumor xenograft sections. **h**, **i** SA-β-gal-positive cells were significantly reduced after pretreatment with BAPTA-AM. However, there was no significant difference in the percentage of senescent cells in control cells upon calcium chelation .**j**, **k** The bar graph showing the quantitative analysis of SA-β-galactosidase-positive cells. **l** Western blot showing that CDKN2A and CDKN1B protein level was significantly up in DOC2B-expressing cells which was significantly reduced upon treatment with BAPTA-AM. **m** Western blot images showing upregulation of CDKN2A and CDKN1A in DOC2B-expressing scrambled cells. Further, calcium depletion significantly reduced CDKN2A and CDKN1A levels. β actin was used as internal control. **n** Bar graph representing the quantitative analysis of CDKN2A, CDKN1A, and CDKN1B before and after calcium chelation (UT, untreated; KD, knockdown; Scr, scrambled)

The gene expression microarray showed DOC2B to module senescence pathway genes (Supplementary Table 5). Upon serum starvation, *DOC2B*-expressing cells showed enlarged flattened senescent morphology with cytoplasmic aggregates when compared to control vector-transfected cells (Fig. 4a). Serum starvation of *DOC2B* overexpressing normal skin fibroblasts also showed senescent morphology with significantly increased senescent cells (Fig. 4a). The SA-β-Gal staining was considerably more in upon ectopic expression of *DOC2B* in SiHa and fibroblast cells (Fig. 4b; *P* < 0.05). The knockdown of *DOC2B* in Cal27 significantly decreased the number of senescence-positive cells (Fig. 4i, k). The western blot analysis showed significantly higher levels of CDKN2A, CDKN1A, and CDKN1B expression in *DOC2B*-overexpressing cells when compared to control vector-transfected cells (Fig. 4c, d). Furthermore, Tri-Methyl-Histone H3 (Lys9) were also elevated in the presence of *DOC2B.* However, the MacroH2A1.2 level was unaltered in control and *DOC2B*-expressing cells (Fig. 4c, d). The immuno-fluorescent analysis showed that the number of cells expressing γ-H2AX and Tri-methyl-histone H3 (Lys9) foci were significantly higher in doxorubicin-treated DOC2B-overexpressing cells (Fig. 4e). Moreover, senescent cells were significantly higher in nude mice receiving DOC2B overexpression cells in comparison to control vector-transfected cells (Fig. 4f, g). These results indicate that DOC2B is associated with induction of senescence. Doxorubicin is well known inducer of senescence and is used as a positive control in senescence experiments (Hu and Zhang 2019; Saleh et al. 2020).

### *DOC2B*-induced senescence and inhibition of EMT requires Ca^2+^

**Fig. 5:**
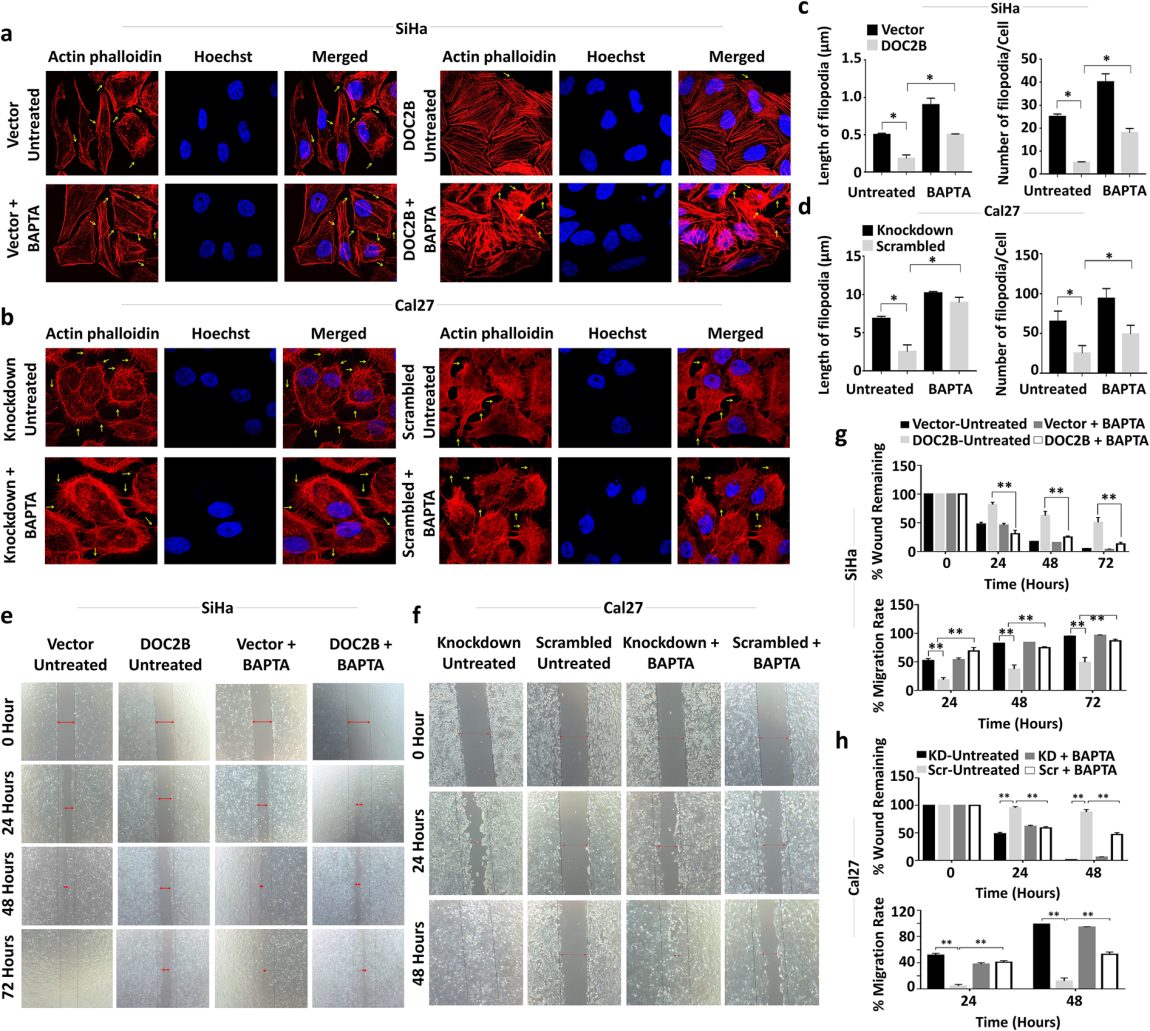
DOC2B-mediated metastatic suppression is calcium-dependent process. For all the experiments cells were pre-treated with BAPTA-AM (10 μM) for 1 h and subsequently used for experiments.**a**, **b** Representative images of actin phalloidin staining of DOC2B over expression and knockdown cells pretreated with BAPTA-AM. Calcium chelation resulted in significant change in actin filament arrangement and loss of cell-to-cell adhesion in *DOC2B* expressing cells in comparison with untreated cells. **c**, **d** Bar graph showing the differential length and number of filopodia in DOC2B-expressing cells before and after calcium chelation**. e**, **f** Representative images showing faster wound closing in DOC2B-expressing cells treated with calcium chelator. **g**, **h** Bar graph representing percentage of wound remaining and percentage migration rate after BAPTA-AM treatment (KD, knockdown; Scr, scrambled).

**Fig. 6:**
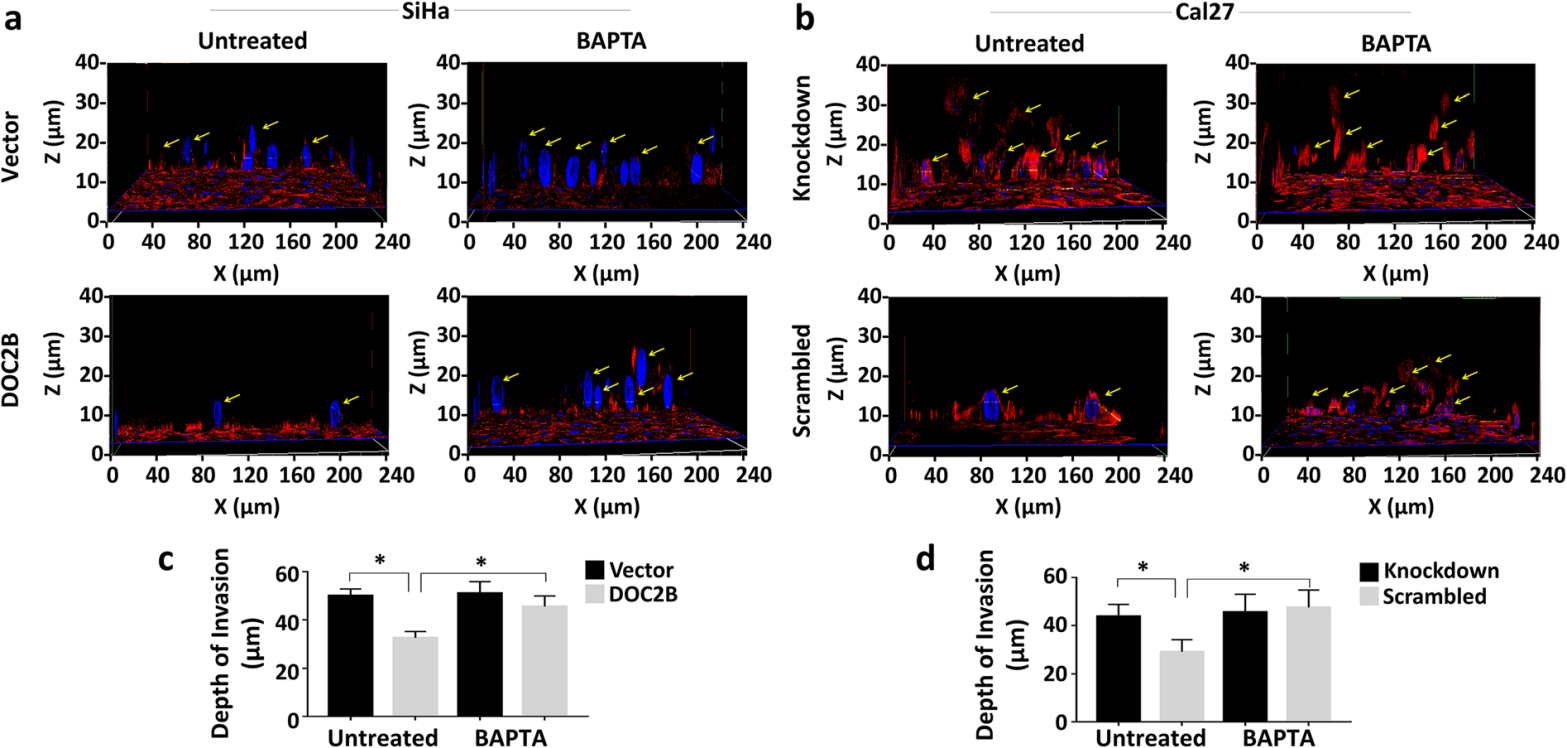
Three-dimensional (3D) invasion Assay. An in vitro 3D invasion assay was performed using DOC2B overexpression and knockdown cells along with respective control cells using collagen I gel. The intracellular calcium depletion was performed by BAPTA-AM treatment and the depth of invasion was analyzed by Z stacking. **a** Representative confocal Z-stacks of BAPTA-AM-treated and untreated cells of overexpression model. **b** Representative confocal Z-stacks of BAPTA-AM-treated and untreated cells of knockdown model. **c**, **d** Bar graph representing the depth of invasion in overexpression and knockdown model respectively. All the experiments were performed in duplicates and repeated three times.

**Fig. 7:**
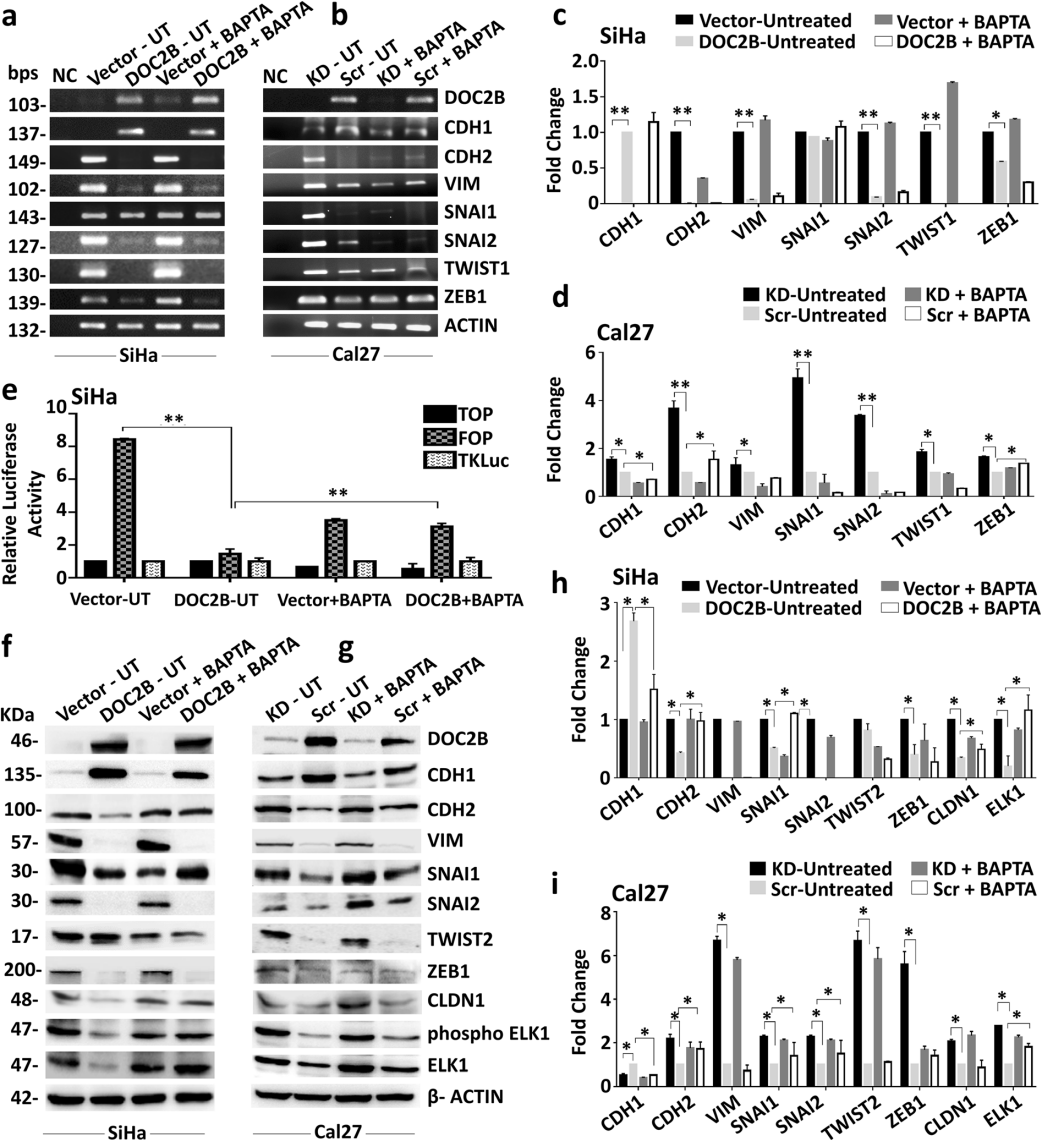
DOC2B-mediated EMT inhibition is partially Ca^2+^ dependent. **a**, **b** The relative mRNA level of CDH1, CDH2, VIM, TW1, TW2, SNAI1, SNAI2 and ZEB1 in DOC2B-expressing cells upon intracellular calcium chelation analyzed by semi–quantitative reverse transcriptase PCR. **c**, **d** Densitometry analysis of semi-quantitative reverse transcriptase PCR results. **e** Bar graph showing quantitative analysis of TOP/FOP activity upon calcium chelation. Pretreatment with BAPTA-AM significantly increased the TOP/FOP activity. **f**, **g** Relative protein levels of CDH1, CDH2, VIM, SNAI1, SNAI2, ZEB1, TWIST2, ELK1, and CLDN1 before and after pretreatment with BAPTA-AM in *DOC2B* overexpression and knockdown cells respectively. Calcium chelation significantly increased the protein levels of CDH2, SNAI1, ELK1, and CLDN1. **h**, **i** Bar graph showing the protein levels of EMT markers in BAPTA-AM-treated and untreated cells (UT, untreated; KD, knockdown; Scr, scrambled). All the experiments were performed in duplicated and repeated three times. The results were analyzed by Student’s *t* test. *P < 0.05 indicates statistical significance

DOC2B is a calcium-dependent protein and many of its functions require calcium. Hence, we next investigated the contribution of intracellular Ca^2+^ to *DOC2B*-induced senescence and inhibition of EMT by pre-treating the cells with BAPTA-AM. BAPTA-AM is used as a cell permeable intracellular Ca^2+^ chelator (Chen et al. 2018). Intracellular calcium depletion significantly reduced the senescent positive cells as well as the expression of CDKN2A and CDKN1B in *DOC2B* expressing cells (Fig. 4h, j, l and n). Among the knockdown models, scrambled Cal27 showed a significantly higher number of senescent cells as opposed to DOC2B knockdown cells. Calcium depletion by BAPTA-AM reduced the senescent positive cells in *DOC2B*-expressing scrambled cells (Fig. 4i, k) and CDKN2A and CDKN1A levels (Fig. 4m, n). The actin cytoskeleton was disorganized upon pre-treatment with BAPTA-AM in *DOC2B*-expressing cells (Fig. 5a, b). The BAPTA-AM treatment significantly increased the number and length of filopodia in *DOC2B*-expressing cells in both overexpression and knockdown models (Fig. 5c, d). Calcium chelation significantly enhanced cell migration in both over expression and knockdown models (Fig. 5e–h). The role of intracellular calcium in DOC2B*-*mediated invasion suppression was investigated by a 3D collagen I invasion assay. The presence of DOC2B significantly inhibited invasion of SiHa and Cal27 cells compared to respective control cells (Fig. 6a, b). The intracellular calcium depletion using BAPTA-AM enhanced invasiveness of DOC2B-expressing cells in both overexpression and knockdown models (Fig. 6c, d). Our findings indicate that intracellular calcium is required for DOC2B-mediated invasion suppression. Among the genes tested for EMT, calcium depletion slightly elevated the mRNA levels of *SNAI1* and *SNAI2* in *DOC2B*-expressing cells (Fig. 7a–d). CDH2, SNAI1, CLDN1, and p-ELK1 protein levels were significantly elevated upon pre-treatment with BAPTA-AM in DOC2B-overexpressing cells (Fig. 7f–i). Interestingly, the levels of CDH1 and TWIST2 were slightly reduced upon calcium depletion. Furthermore, calcium depletion also significantly increased the TOP/FOP reporter activity in *DOC2B*-overexpressing cells (Fig. 7e). Collectively, these results suggest the role of intracellular Ca^2+^ in *DOC2B*-induced senescence and inhibition of EMT.

### DOC2B localizes with CDH1 and CTNNB1 in the plasma membrane

**Fig. 8:**
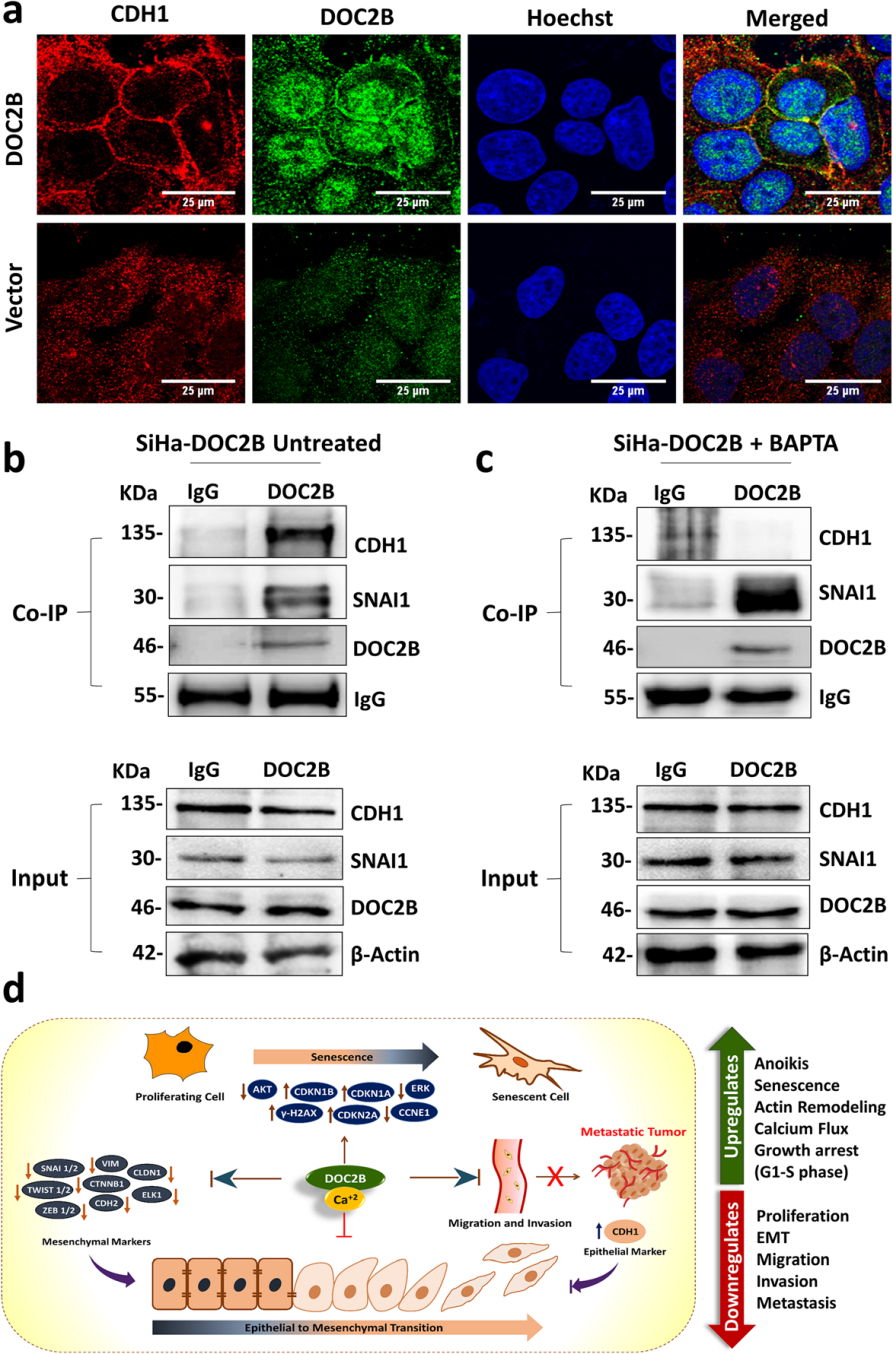
DOC2B interacts with CDH1 and SNAI1 in SiHa. **a** Representative confocal images of localization of DOC2B and CDH1 in control and DOC2B-overexpressing cells. CDH1 was co-localized along with DOC2B in the plasma membrane. **b** Co-IP of endogenous DOC2B with CDH1 and SNAI1 in SiHa cells. DOC2B was immunoprecipitated, and the amount of CDH1 and SNAI1 bound to DOC2B was determined using immunoblot with anti-CDH1 and anti-SNAI1antibody. **c** Pretreatment of DOC2B-expressing SiHa cells with BAPTA-AM abolished the interaction between CDH1 and DOC2B. **d** Proposed model for *DOC2B-*mediated tumor suppressive function. *DOC2B* regulates EMT and senescence process via complex cross talk between Ca^2+^ and multiple pathways involving cell surface receptors (CD55, CD61), signal-transducing molecules (RAS, ERK1/2, AKT1, RAC1, CDC42, ELK1, CCNE, CTNNB1, CDKN2A, CDKN1A, and CDKN1B), and effector molecules (CDH1, VIM, CDH2, TWIST1, TWIST2, SNAIL, SLUG, and ZEB1) resulting in senescence and inhibition of EMT.

The co-localization of DOC2B, CDH1, and CTNNB1 was assessed by immunofluorescence and confocal microscopy. The confocal images showed localization of DOC2B with CDH1 and CTNNB1 in the plasma membrane in DOC2B-overexpressing cells. In contrast, both CDH1 and DOC2B did not show any localization to the plasma membrane, while CTNNB1 was predominantly localized to the nucleus in control cells (Fig. 8a, Supplementary Fig. 6G).

### DOC2B physically interacts with CDH1 in a calcium-dependent manner

We next interrogated the physical interaction between DOC2B, CDH1, and SNAI1 by Co-IP experiment. The Co-IP experiment showed that DOC2B physically interacts with CDH1 and SNAI1 (Fig. 8b). Intracellular calcium depletion abolished the interaction between DOC2B and CDH1 (Fig. 8c). These results collectively showed that DOC2B and CDH1 interaction requires calcium.

### *DOC2B* represses β-catenin-induced TCF activation

Gene expression microarray showed DOC2B to target Wnt signaling (Supplementary Table 6). The active form of CTNNB1 was downregulated in the presence of DOC2B without any change in its mRNA level (Supplementary Fig. 6A–D). GSK3α/β protein, a member of the CTNNB1 degradation complex, was also upregulated in the presence of *DOC2B* (Supplementary Fig. 6B, D). The luciferase assay revealed that the endogenous level of TCF activity was repressed by *DOC2B* (Supplementary Fig. 6E and 6F).

It has been reported that S33Y, a mutant form of CTNNB1, binds to TCF and is not degraded by the CTNNB1 degradation complex, leading to continuous activity of CTNNB1 (Akimoto et al. 2005). Here, we investigated whether *DOC2B* inhibits TCF activity even in the presence of the active S33Y mutant CTNNB1 by co-transfecting the TOP-FLASH vector along with wild type (WT) or mutant CTNNB1 (S33Y) separately into *DOC2B* overexpression or knockdown cells. Irrespective of whether the cells contain wild type or mutant CTNNB1 (S33Y), TCF activity was reduced in the presence of *DOC2B*.

## Discussion

While EMT in tumor cells may lead to invasive and metastatic phenotypes, senescence as a phenomenon has much wider effects. Previously, we reported that *DOC2B* is a methylation-regulated gene, silenced in cervical cancer, and its downregulation is important for the acquisition of key biological characteristics of cervical cancer cells (Patsialou et al. 2012). A study by Patsialou et al. (2012) has listed DOC2B as one of the downregulated genes in migratory breast cancer cells, suggesting its role as a negative regulator of cancer (Patsialou et al. 2012). Among various cancers, DOC2B and its functions were primarily studied in cervical cancer; however, causal biological mechanisms and cell signaling pathways leading to functional perturbations were elusive. We showed that DOC2B upregulation inhibits metastasis in cervical cancer via two distinct mechanisms: activation of senescence and inhibition of EMT. To support these, we present evidence to show that (a) inhibition of *DOC2B* in tumor models leads to more aggressive behavior with the concomitant enhancement in mesenchymal markers expression, and (b) expression or reactivation of DOC2B in tumor cells leads to inhibition of growth, invasion, EMT, and induction of SASP and associated markers.

The relationship between senescence and EMT is a context-dependent complex process (Yang and Weinberg 2008). Previous studies have indicated the existence of senescence-EMT cross talk as a mechanism of metastatic suppression. Activation of EMT is positively correlated with metastasis and tumor progression. Furthermore, induction of senescence is negatively correlated with metastasis. Our study for the first time reports that DOC2B can induce senescence, and inhibition of EMT is a Ca^2+^dependent process. This observation is supported by our calcium depletion experiments which showed that treatment with BAPTA-AM (i) attenuated senescence via downregulation of senescence markers (CDKN2A, CDKN1A and CDKN1B) and (ii) activated EMT by enhancing the expression of CDH2, SNAI1, ELK1 and CLDN1, and slight reduction of CDH1. We show for the first time that DOC2B is a metastatic suppressor and propose that reactivation of DOC2B could be used to control cancer metastasis.

It is well known that p53 and/or p16/pRb pathways play critical roles in the establishment and maintenance of senescence (Muñoz-Espín and Serrano 2014). Both CDKN2A and CDKN1A function together to maintain the hypo-phosphorylated state of pRb to induce senescence (Wen et al. 2014). In our study, senescence induction upon *DOC2B* restoration depends on CDKN2A and CDKN1B. During senescence, CCNE expression is downregulated by Cip/Kip family of proteins (CDKN2A and CDKN1B) preventing CDK2-CCNE interaction leading to pRb hypo-phosphorylation and senescence induction (Stein et al. 1999). Over expression of CCNE has been shown to be associated with tumor progression (Alexander et al. 2017). Thus, the induction of senescence in the presence of DOC2B might be due to inhibition of CCNE by CDKN2A and CDKN1B.

EMT is the major mechanism of metastasis and regulates many aspects of tumor progression (Larue and Bellacosa 2005; Qureshi et al. 2015; Wu et al. 2016). Overexpression of mesenchymal markers along with EMT-TFs is an important event for invasion and metastasis. Activated CTNNB1, through signalling events initiated by phosphorylation of AKT, translocates to the nucleus and initiates the expression of downstream targets such as CCND1 and CCNE, TWIST, SNAIL, MMPs, C-MYC, and several others rendering tumor cells more invasive (He et al. 2009; Karim et al. 2004; Klaus and Birchmeier 2008; Yook et al. 2005). Loss of CDH1, a central step in EMT, is mediated by SNAILs, ZEBs, and KLF8 either by binding directly to *CDH1* promoter or indirectly through their interaction with *TWIST, TCF4, SIX1* and *FOXC2* mediated via Wnt/β-catenin, TGF-β, EGF, HGF, and Notch signalling (Lamouille et al. 2014; Yang and Weinberg 2008). Downregulation of EMT-TFs upon *DOC2B* overexpression may be linked to Ras-MAPK, Wnt/β-catenin and PI3K-AKT pathways as these pathways have shown to activate EMT–TFs leading to downregulation of *CDH1* along with simultaneous activation of *CDH2* and *VIM* resulting in invasion and metastasis in numerous cancers (Hong et al. 2011; Larue and Bellacosa 2005). Furthermore, these pathways are downregulated in cells undergoing senescence (Smit and Peeper 2010; Ye et al. 2007). The present study is the first report to show the link between DOC2B and RAS, RAC1, and CDC42. Our findings show that the presence of DOC2B significantly reduces the active form of RAS, RAC1, and CDC42. The active form of RAS (Tripathi and Garg 2018), RAC1 (Zhou et al. 2016), and CDC42 (Ungefroren et al. 2018) has been reported to facilitate the acquisition of various cancer hallmarks, notably proliferation, migration, invasion, and metastasis via upregulation of EMT. Besides, RAS, RAC1, and CDC42 also reported as inhibitors of senescence. Moreover, RAS, RAC1, and CDC42 play an active role in actin polymerization and turnover, which significantly impact filopodia and lamellipodia formation and cell motility (Sit and Manser 2011). Besides, intracellular Ca^2^^+^ and calcium signaling play an active role in governing the expression and function of RAS, RAC1, and CDC42 (Aspenström 2004; Price et al. 2003). These data collectively suggest that DOC2B may be linked with RAS, RAC1, and CDC42 via intercellular Ca^2+^. However, more detailed investigations are required before further conclusions are drawn.

We showed that DOC2B interacts with CDH1and SNAI1 in SiHa cells. Interestingly, calcium depletion completely abolishes the DOC2B-CDH1 interaction, suggesting this interaction as a calcium-dependent event. In future studies, we intended to precisely understand the role of intracellular calcium in DOC2B-CDH1 interaction and identify the critical regions facilitating the interaction and its contribution to metastatic suppression. Decreased expression of stemness markers such as CD55 and CD61 in *DOC2B*-overexpressing cells also suggests inhibition of a more aggressive phenotypes (Jay et al. 2004; Patki et al. 2010). CD61 is a well-known member of integrin family with diverse role in neoplastic transformation, specifically for its role in EMT in numerous cancers (Deep et al. 2014; Lei et al. 2011). Many studies have reported the upregulation of CD61 during mesenchymal transition and its minimal expression in the normal epithelial cells (Mamuya and Duncan 2012). Further, CD61 is associated with metastasis in cutaneous melanoma (Othman et al. 2007; Sominidi-Damodaran et al. 2016) and breast cancer (Galliher and Schiemann 2006). CD55 is overexpressed in many of the in situ tumor cells and is further enhanced in the presence of HPV E6 protein and is responsible for radio resistance and cancer aggressiveness (Leung et al. 2018). Silencing of CD51 expression was shown to attenuate cancer growth in prostate cancer condition (Loberg et al. 2006). A recent study have demonstrated the anti-proliferative and anti-metastatic role of anti-CD55 monoclonal antibody in colorectal cancer cells (Dho et al. 2019). These data collectively suggest that DOC2B inhibits metastasis by targeting the EMT-senescence axis.

We have investigated the role of *DOC2B* in regulating Wnt signaling as it is reported to confer EMT and inhibition of senescence. Gene expression microarray data showed the co-expression of genes related to senescence, EMT, and Wnt signaling as enriched upon perturbed DOC2B expression. AKT is a key signal transducer during PI3K signaling, and its aberrant activation is well reported in different tumors. Phosphorylation at Thr308 and Ser473 residues result in the activation of AKT. Several studies have reported the significance of AKT activation in cancer cell invasion, motility, and actin organization (Chin and Toker 2009). p38MAPK modulated cell migration by inducing MAPK-activated protein kinase 2/3 (MAPKAP 2/3) phosphorylation, that in turn is critical for the directionality of migration (Huang 2004). The reduced level of active forms of CTNNB1, AKT1, ERK1/2, RAS, RAC1, CDC42, and inhibition of TCF activity upon manipulation of DOC2B may also contribute to the anti-EMT and pro-senescence function of DOC2B.

*DOC2B* requires Ca^2+^ for many of its functions. To directly explore the role of Ca^2+^ in *DOC2B*-induced senescence and inhibition of EMT, cells were pretreated with BAPTA-AM and assessed for expression of senescence and EMT markers. Pretreatment of DOC2B-expressing cells with BAPTA-AM altered actin cytoskeleton network, morphological changes, and loss of cell to cell adhesion. We showed that senescence induced by *DOC2B* was significantly reduced upon treatment with BAPTA-AM. Furthermore, we have also identified CDH2, SNAI1, CLDN1, and p-ELK1 were also upregulated upon pretreatment with BAPTA-AM. Furthermore, the TCF activity significantly increased upon pretreatment with BAPTA-AM. Our research findings propose the critical role of Ca^2+^ in controlling *DOC2B*-induced senescence and inhibition of EMT.

## Conclusion

Our findings suggest that DOC2B acts as a potent tumor growth regulator and confers metastatic resistance via DOC2B-calcium-EMT-senescence axis in cervical cancer. Our present study has multiple clinical applications. Primarily, methylation and expression analysis of DOC2B could be used as marker for early diagnosis of cervical cancer. Furthermore, EMT activation has been recognized as a key mechanism in metastasis and therapy resistance. Since DOC2B is a suppressor of EMT, analysis of DOC2B expression may be used as a marker to predict metastasis and therapeutic resistance in cervical cancer. *DOC2B*-induced senescence and the anti-EMT effect are calcium-dependent and important for the prevention of invasive phenotype (Fig. 8d). Collectively, based on the functional role of DOC2B and associated signaling pathways, targeting DOC2B–Calcium–EMT-senescence axis may offer a novel approach for controlling metastasis in cervical cancer.

## Supplementary information

ESM 1 Knockdown of DOC2B in the over expression model activates cancer hallmarks. **A** and **B)** Representative RT-PCR and western blot images showing the knockdown of DOC2B in SiHa-DOC2B cells. **C)** Confocal images showing actin rearrangement in DOC2B-expressing and knockdown cells. **D)** Bright field microscopic images of SiHa-DOC2B and DOC2B knockdown cells. **E)** DOC2B knockdown in SiHa-DOC2B cells significantly enhanced cell proliferation. **F** and **G)** Represents increase in the colony forming ability of DOC2B knockdown SiHa-DOC2B cells. **H, I** and **J)** Silencing of DOC2B in the over expression model significantly elevated cell migration rate. (PNG 2512 kb)

High Resolution Image (TIF 10259 kb)

ESM 2 Gene expression microarray analysis. **A)** Represents the hierarchical clustering of gene expression microarray data performed using HCE3.5 software by Euclidian distance and average linkage method. The upregulated and down regulated genes are represented by red and green colors respectively. **B)** Represents biological pathways significantly enriched in downregulated and upregulated genes upon DOC2B overexpression in SiHa cells respectively. **C)** Represents biological pathways significantly enriched in downregulated and upregulated genes upon DOC2B knockdown in Cal27 cells respectively. (PNG 620 kb)

High Resolution Image (TIF 2599 kb)

ESM 3 Gene ontology, interaction and pathways regulated by *DOC2B* **A)** Represents the bar graphs of gene ontology terms (Molecular functions, Biological process and cellular components) significantly enriched in downregulated and upregulated genes upon DOC2B overexpression in SiHa cells respectively. **B)** Represents the pie chart of gene ontology terms (Molecular functions, Biological process and cellular components) significantly enriched in downregulated and upregulated genes upon DOC2B knockdown in Cal27 cells respectively. The pathway enrichment analysis was performed using Enricher (http://amp.pharm.mssm.edu/Enrichr/). (PNG 1363 kb)

High Resolution Image (TIF 9210 kb) (TIF 3094 kb)

ESM 4 Gene ontology and pathways regulated by common genes identified in overlapping analysis. **A-D)** Representative bar graphs of enriched pathways and gene ontology terms (Molecular functions, Biological process and cellular components) in common genes identified. The pathway enrichment analysis was performed using Enricher (http://amp.pharm.mssm.edu/Enrichr/). (PNG 885 kb)

High Resolution Image (TIF 3094 kb) (TIF 9210 kb)

ESM 5 Semi quantitative RT-PCR showing the expression levels of DOC2B and its target genes in metastatic liver tissue in DOC2B overexpression model. (PNG 638 kb)

High Resolution Image (TIF 1190 kb)

ESM 6 *DOC2B* suppresses β-catenin signaling and β-catenin induced TCF/LEF activity. **A** and **C)** Relative expression levels of CTNNB1 in the *DOC2B* overexpressing and knockdown cells when compared to their respective controls as measured by semi-quantitative RT-PCR. **B** and **D)** Western blot analysis for CTNNB1 and GSK3A/B in *DOC2B* overexpression and knockdown cells respectively. **E** and **F)** Dual luciferase assay showing the effect of *DOC2B* on TOPFLASH / FOPFLASH activity in *DOC2B* overexpressing SiHa cells and *DOC2B* knockdown Cal27 cells respectively. TCF activity was repressed from 3.2 ± 0.32 to 1.04 ± 0.43 fold in the presence of *DOC2B* when compared to control SiHa cells (P = 0.02). In contrast, the knockdown of *DOC2B* enhanced the TCF activity from 3.72 ± 0.23 to 8.15 ± 0.44 fold (P = 0.006). Cells were co-transfected with TOPFLASH reporter and/or FOPFLASH reporter plasmid with mutant (S33Y) or wild-type (WT) CTNNB1 expression plasmid. The values were normalized to an internal Renilla luciferase. *DOC2B* significantly inhibits β-catenin transcriptional activity. In control cells, the TCF activity was found to be 4.2 ± 0.25 and 14.6 ± 0.58 fold in cells transfected with wild type and mutant β-catenin. In contrast, TCF activity was found to be 1.1 ± 0.386 and 4.8 ± 0.78 fold in *DOC2B* expressing SiHa cells transfected with wild type and mutant CTNNB1 respectively. The transfection of wild type and mutant CTNNB1 enhanced the TCF activity (16.1 ± 0.784 fold and 22 ± 0.41 fold) in cells transfected with wild type and mutant CTNNB1 in *DOC2B* knock down Cal27 cells as opposed to scrambled vector transfected Cal27 cells (3.86 ± 0.29 fold and 1.4 ± 0.19 fold in cells transfected with wild type and mutant CTNNB1) (P < 0.05). **G)** Representative confocal images of localization of DOC2B and CTNNB1 in control and DOC2B-overexpressing cells. CTNNB1 was co-localized along with DOC2B in the plasma membrane. DOC2B expression inhibited the nuclear translocation of CTNNB1. Results of each experiment are presented as the mean ± SD for three independent experiments. *P < 0.05 indicates statistical significance. (PNG 1966 kb)

High Resolution Image (TIF 4552 kb)

ESM 7 Top differentially expressed pathways enriched upon *DOC2B* overexpression (XLSX 11 kb)

ESM 8 Top differentially expressed pathways enriched upon *DOC2B* knockdown (XLSX 11 kb)

ESM 9 Common molecular players identified in overlapping analysis between up and down regulated genes in DOC2B over expression and knockdown models (XLSX 12 kb)

ESM 10 Genes related to EMT (XLSX 11 kb)

ESM 11 Genes related to Senescence (XLSX 9 kb)

ESM 12 Genes related to Wnt signaling (XLSX 10 kb)

## Data Availability

The authors declare that the data supporting the findings of this study are available within the paper and its Supplementary information files. All other data are available from the corresponding author upon reasonable request.
