# Supplementary figures and images for: Metastatic suppression by DOC2B is mediated by inhibition of epithelial-mesenchymal transition and induction of senescence
Source: Cell Biol Toxicol. 2021 Mar 24;38(2):237–58. doi: 10.1007/s10565-021-09598-w (PMC8986756; doi:10.1007/s10565-021-09598-w)

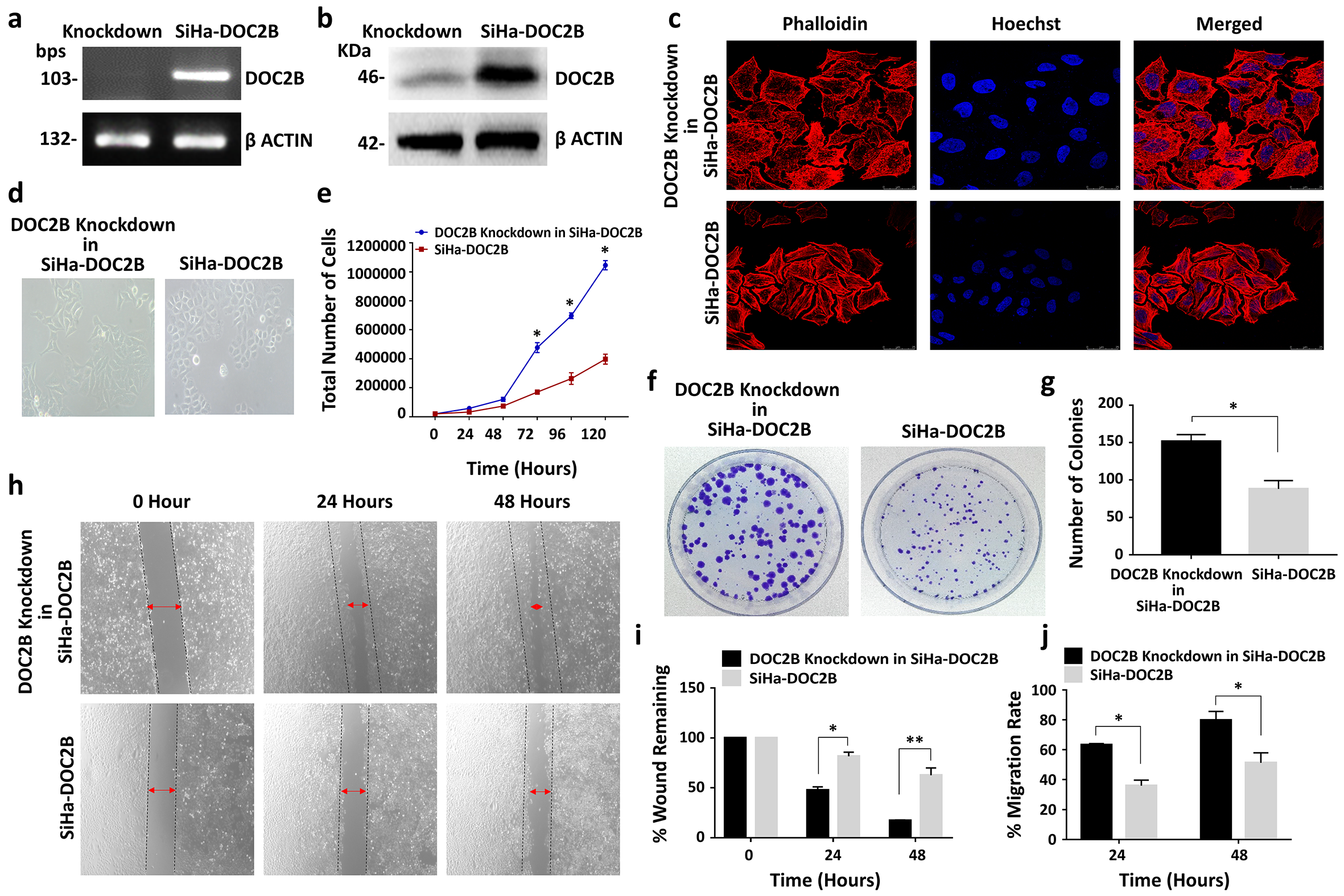

Supplement: Supplementary file 1 — Knockdown of DOC2B in the over expression model activates cancer hallmarks. A and B) Representative RT-PCR and western blot images showing the knockdown of DOC2B in SiHa-DOC2B cells. C) Confocal images showing actin rearrangement in DOC2B-expressing and knockdown cells. D) Bright field microscopic images of SiHa-DOC2B and DOC2B knockdown cells. E) DOC2B knockdown in SiHa-DOC2B cells significantly enhanced cell proliferation. F and G) Represents increase in the colony forming ability of DOC2B knockdown SiHa-DOC2B cells. H, I and J) Silencing of DOC2B in the over expression model significantly elevated cell migration rate. (PNG 2512 kb) [file 10565_2021_9598_Fig9_ESM.png]

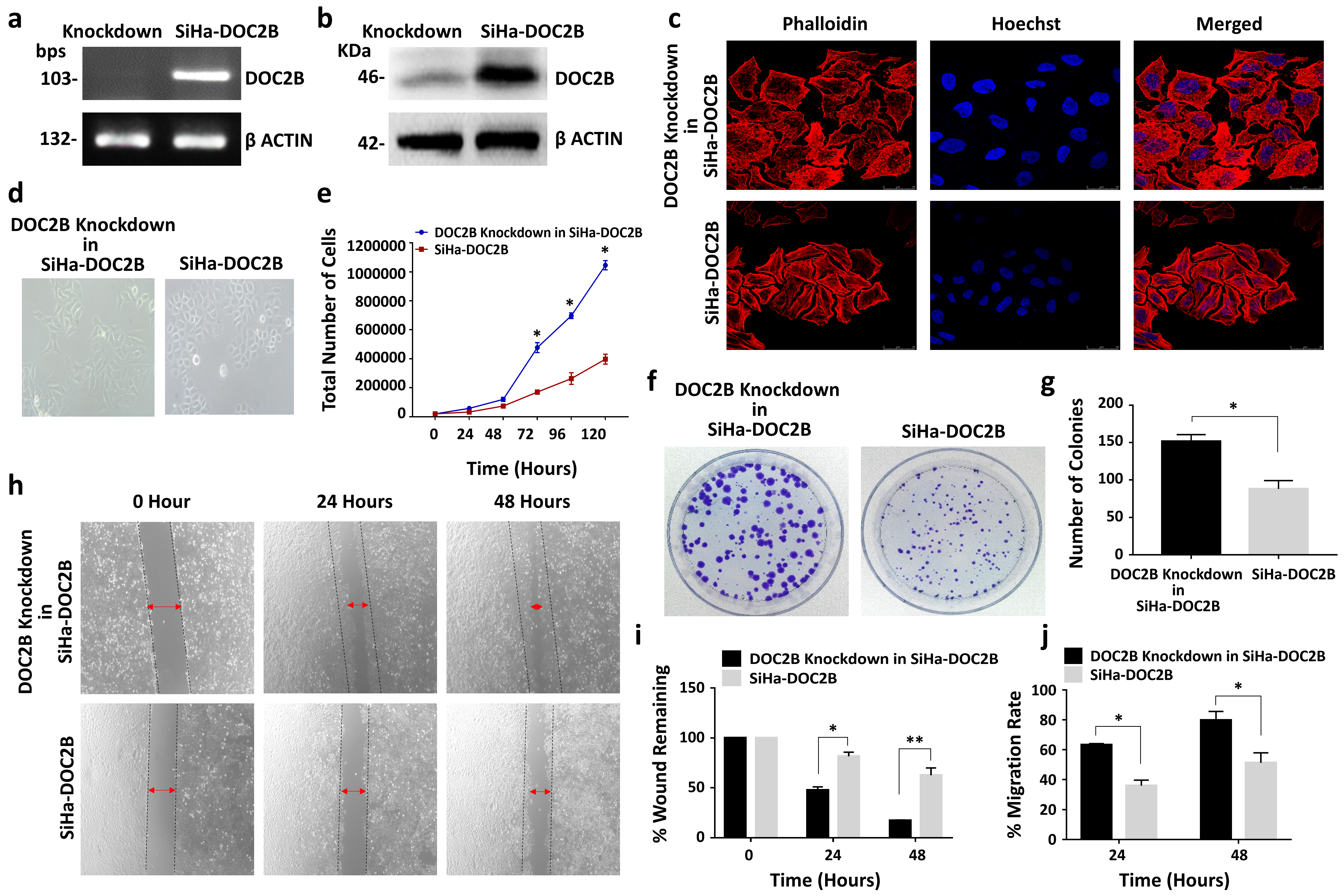

Supplement: Supplementary file 2 — High Resolution Image (TIF 10259 kb) [file 10565_2021_9598_MOESM1_ESM.tif]

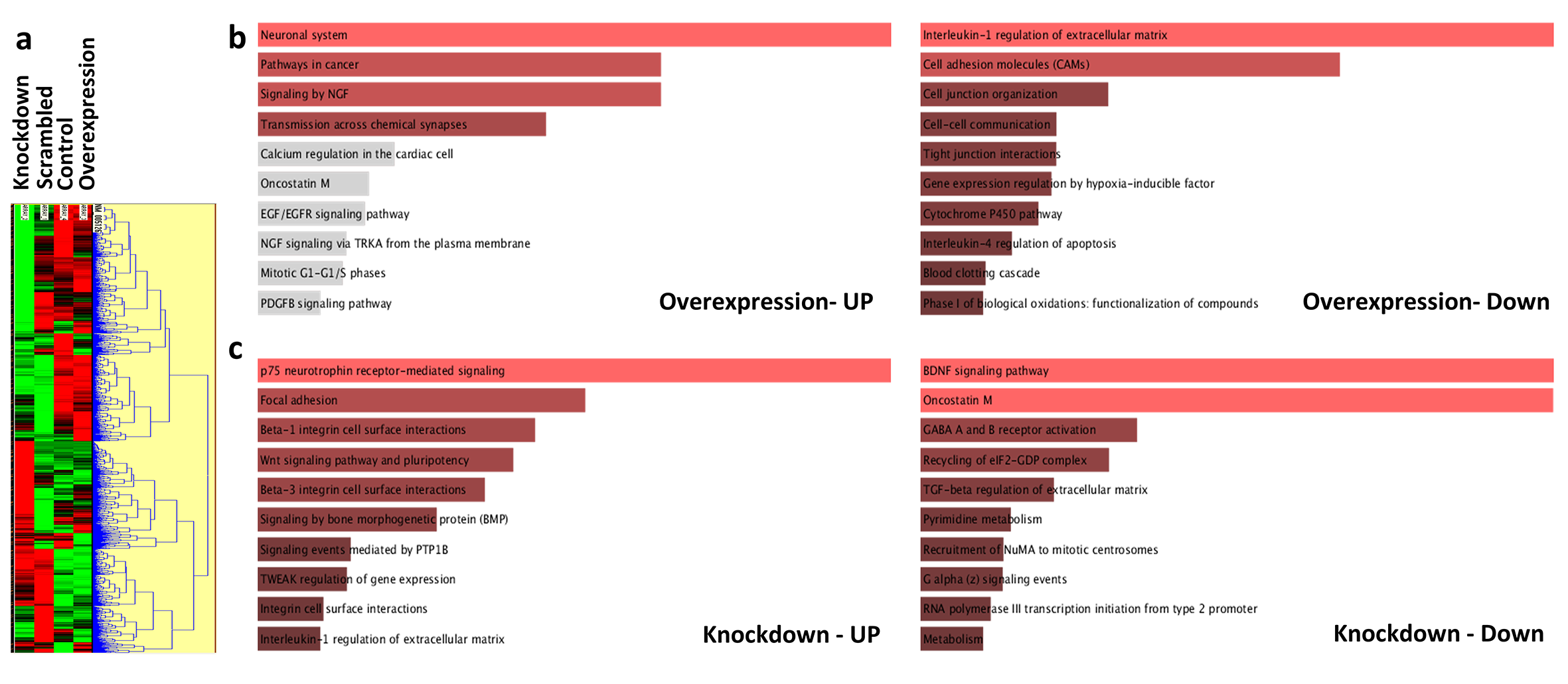

Supplement: Supplementary file 3 — Gene expression microarray analysis. A) Represents the hierarchical clustering of gene expression microarray data performed using HCE3.5 software by Euclidian distance and average linkage method. The upregulated and down regulated genes are represented by red and green colors respectively. B) Represents biological pathways significantly enriched in downregulated and upregulated genes upon DOC2B overexpression in SiHa cells respectively. C) Represents biological pathways significantly enriched in downregulated and upregulated genes upon DOC2B knockdown in Cal27 cells respectively. (PNG 620 kb) [file 10565_2021_9598_Fig10_ESM.png]

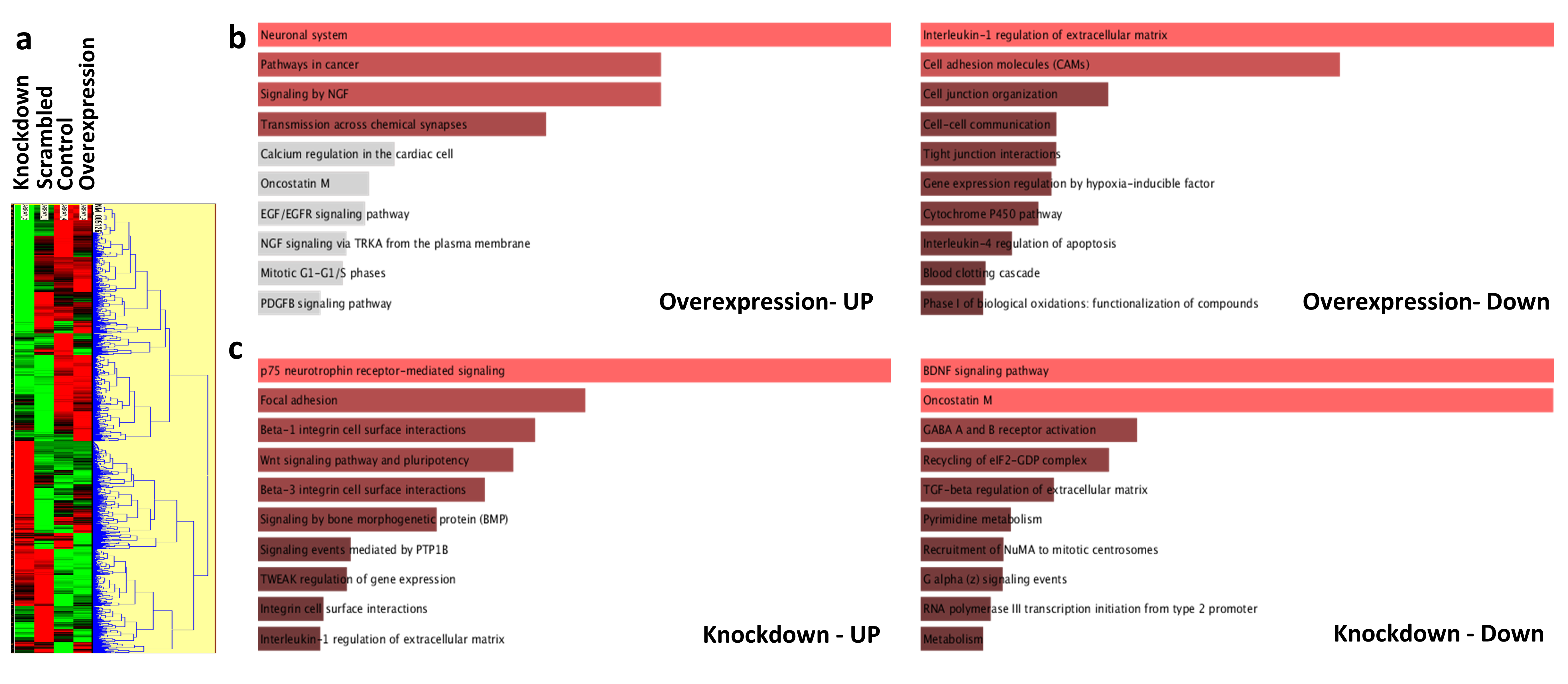

Supplement: Supplementary file 4 — High Resolution Image (TIF 2599 kb) [file 10565_2021_9598_MOESM2_ESM.tif]

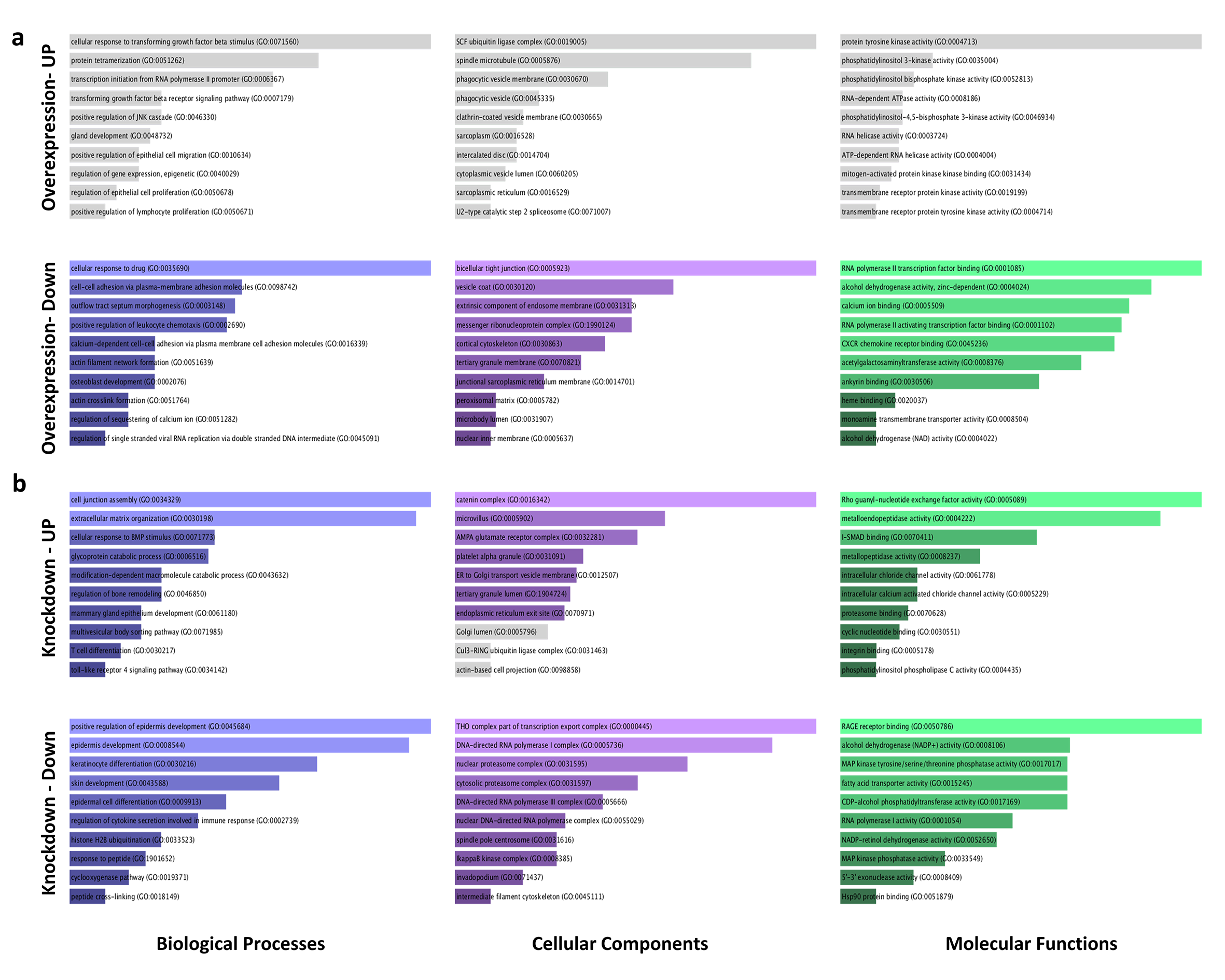

Supplement: Supplementary file 5 — Gene ontology, interaction and pathways regulated by DOC2B A) Represents the bar graphs of gene ontology terms (Molecular functions, Biological process and cellular components) significantly enriched in downregulated and upregulated genes upon DOC2B overexpression in SiHa cells respectively. B) Represents the pie chart of gene ontology terms (Molecular functions, Biological process and cellular components) significantly enriched in downregulated and upregulated genes upon DOC2B knockdown in Cal27 cells respectively. The pathway enrichment analysis was performed using Enricher (http://amp.pharm.mssm.edu/Enrichr/). (PNG 1363 kb) [file 10565_2021_9598_Fig11_ESM.png]

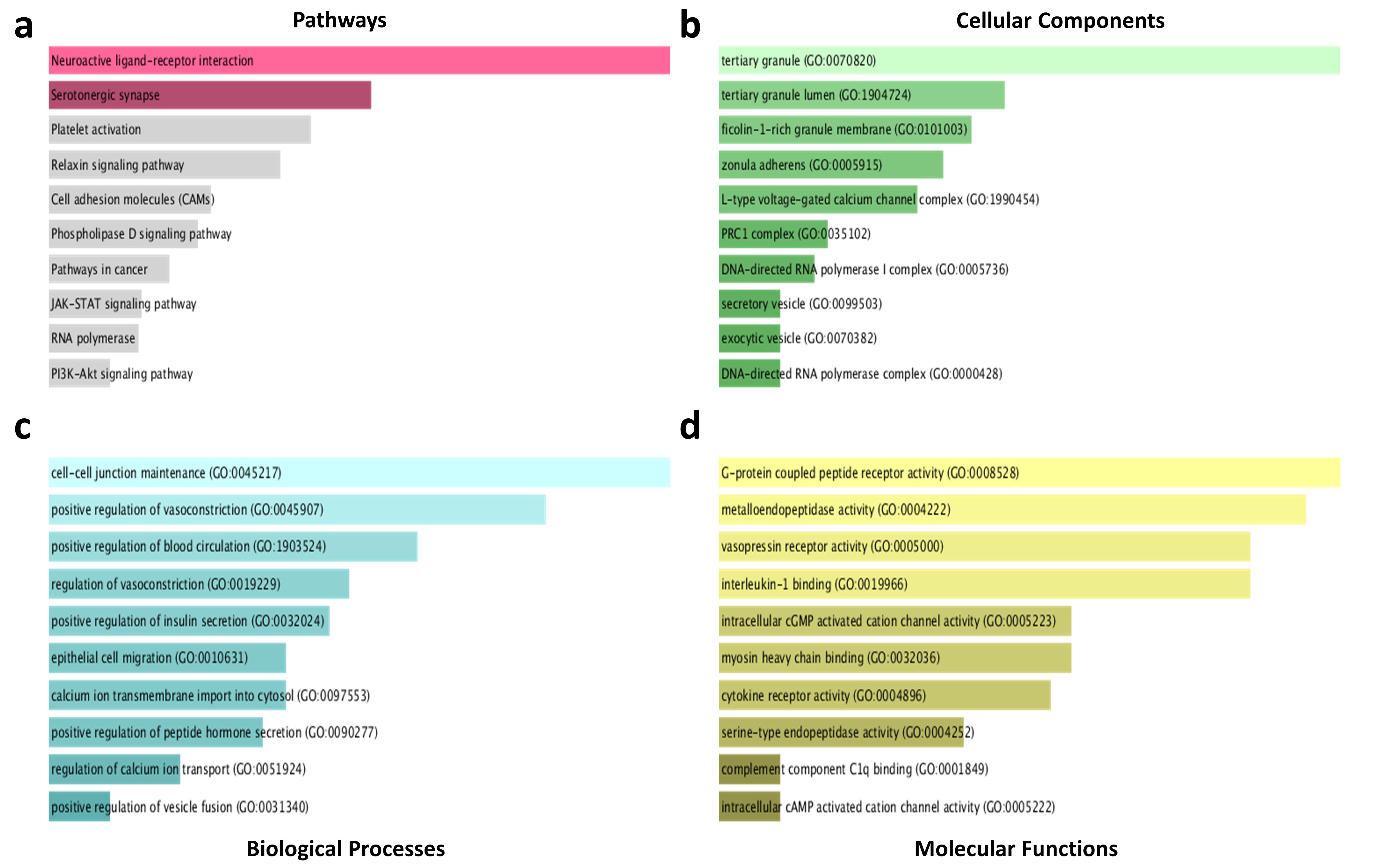

Supplement: Supplementary file 6 — High Resolution Image (TIF 9210 kb) (TIF 3094 kb) [file 10565_2021_9598_MOESM4_ESM.tif]

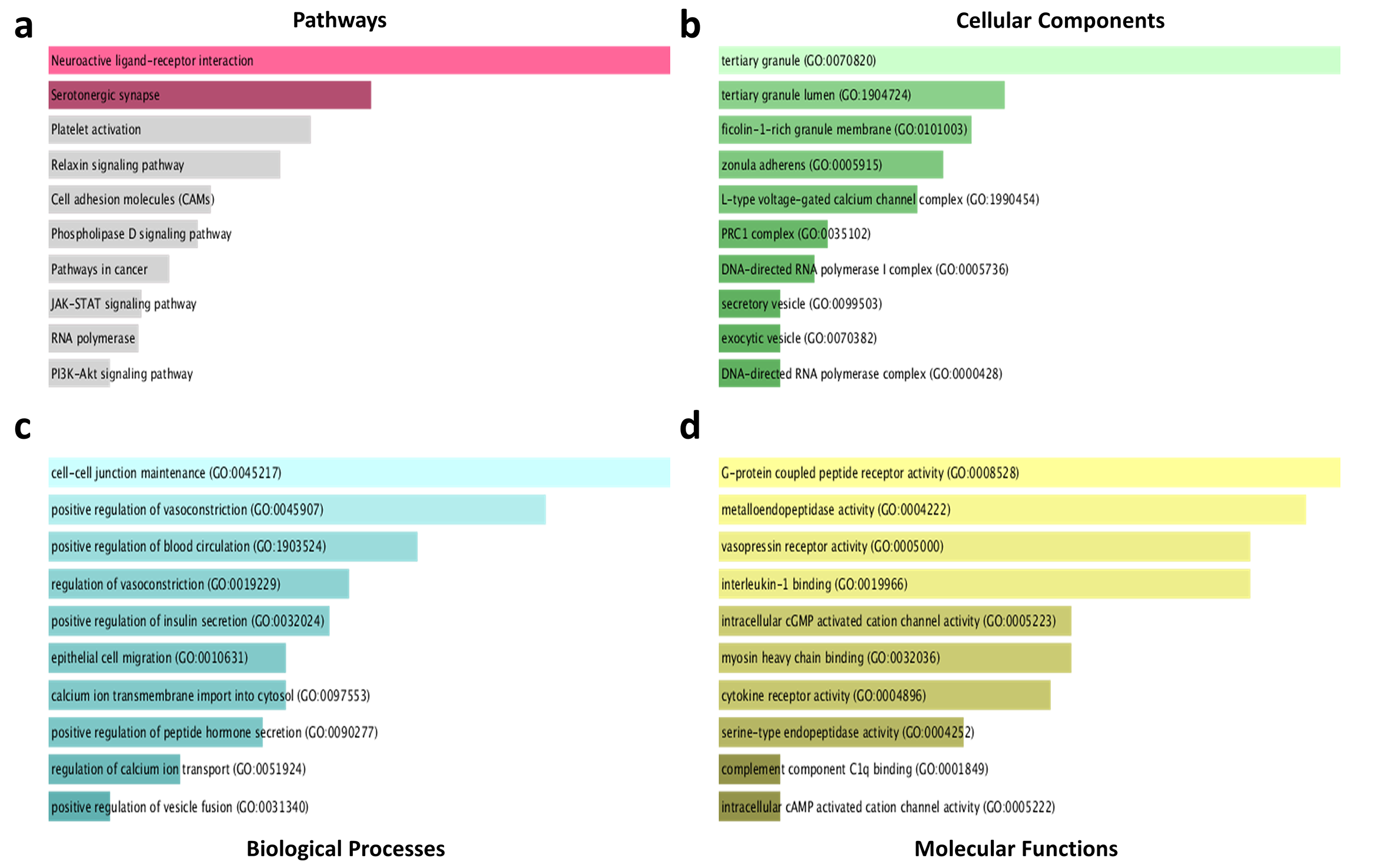

Supplement: Supplementary file 7 — Gene ontology and pathways regulated by common genes identified in overlapping analysis. A-D) Representative bar graphs of enriched pathways and gene ontology terms (Molecular functions, Biological process and cellular components) in common genes identified. The pathway enrichment analysis was performed using Enricher (http://amp.pharm.mssm.edu/Enrichr/). (PNG 885 kb) [file 10565_2021_9598_Fig12_ESM.png]

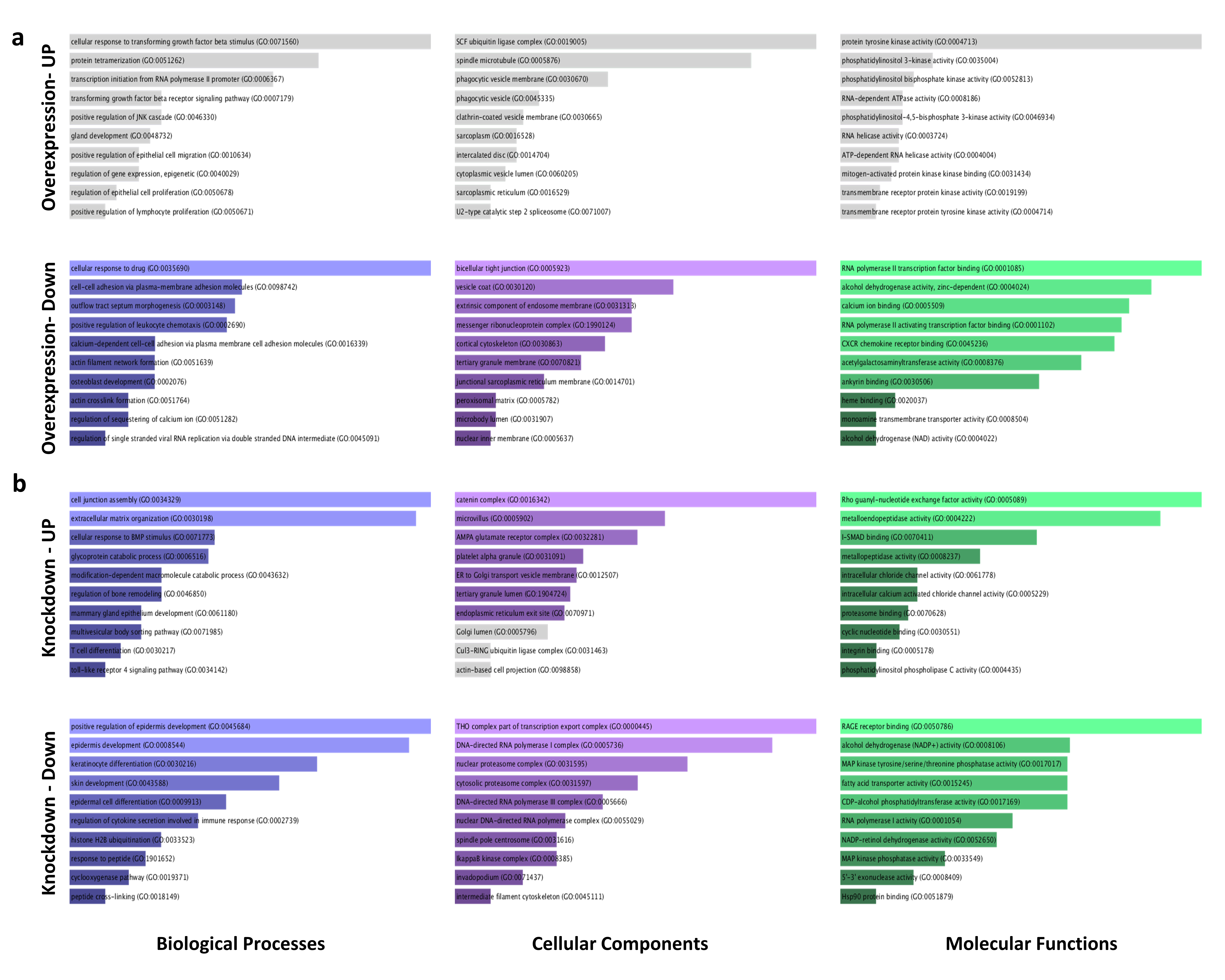

Supplement: Supplementary file 8 — High Resolution Image (TIF 3094 kb) (TIF 9210 kb) [file 10565_2021_9598_MOESM3_ESM.tif]

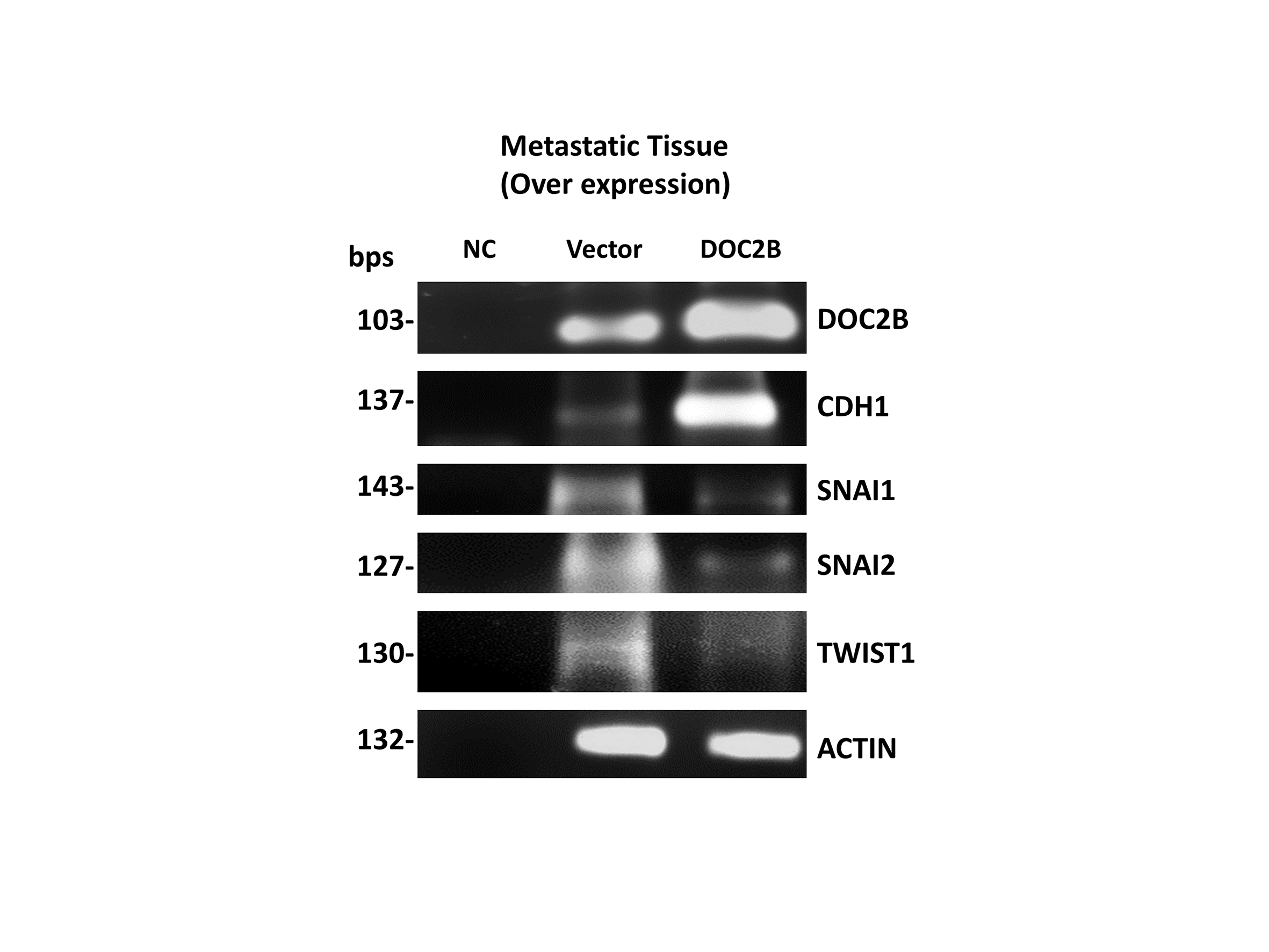

Supplement: Supplementary file 9 — Semi quantitative RT-PCR showing the expression levels of DOC2B and its target genes in metastatic liver tissue in DOC2B overexpression model. (PNG 638 kb) [file 10565_2021_9598_Fig13_ESM.png]

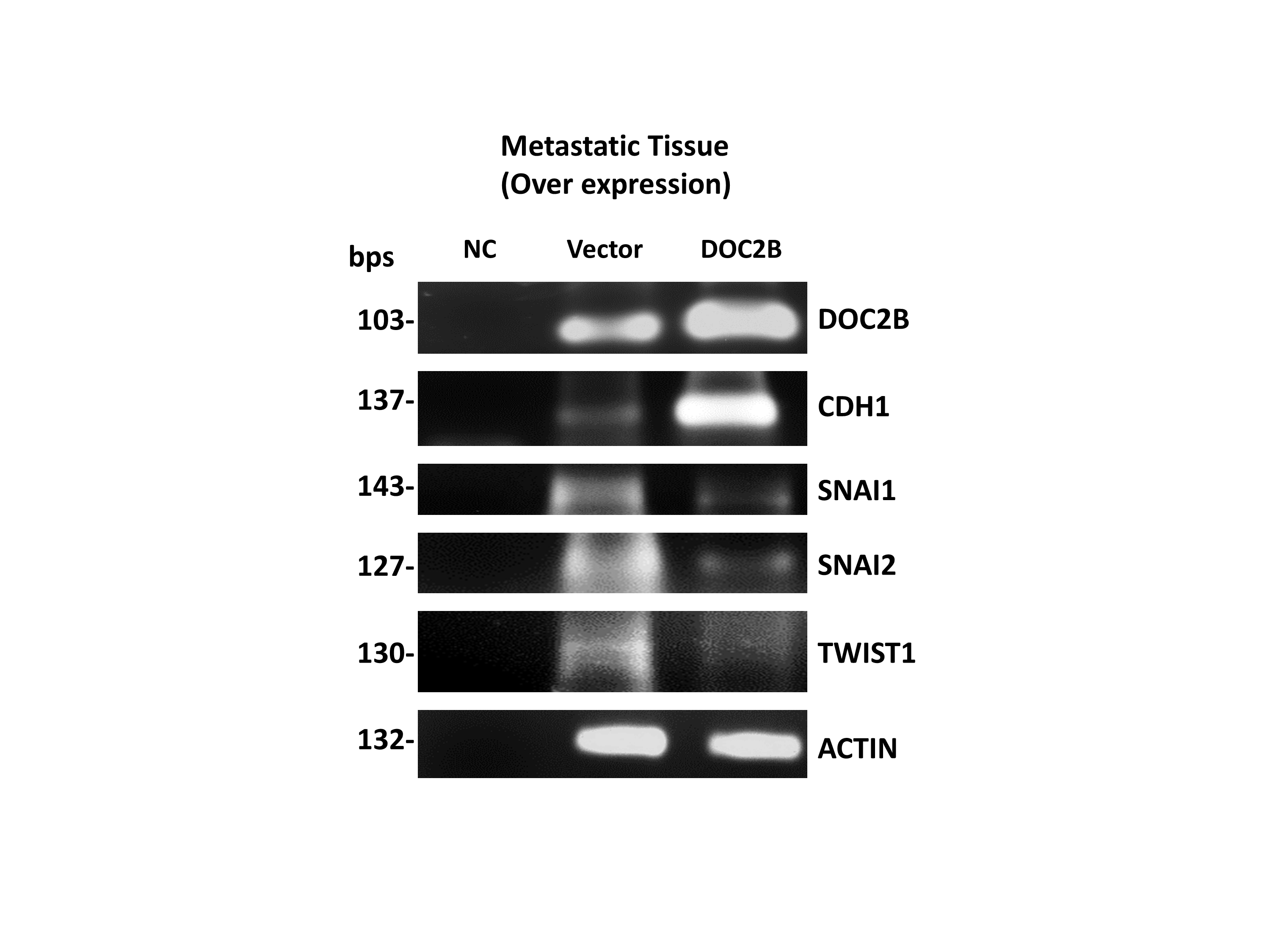

Supplement: Supplementary file 10 — High Resolution Image (TIF 1190 kb) [file 10565_2021_9598_MOESM5_ESM.tif]

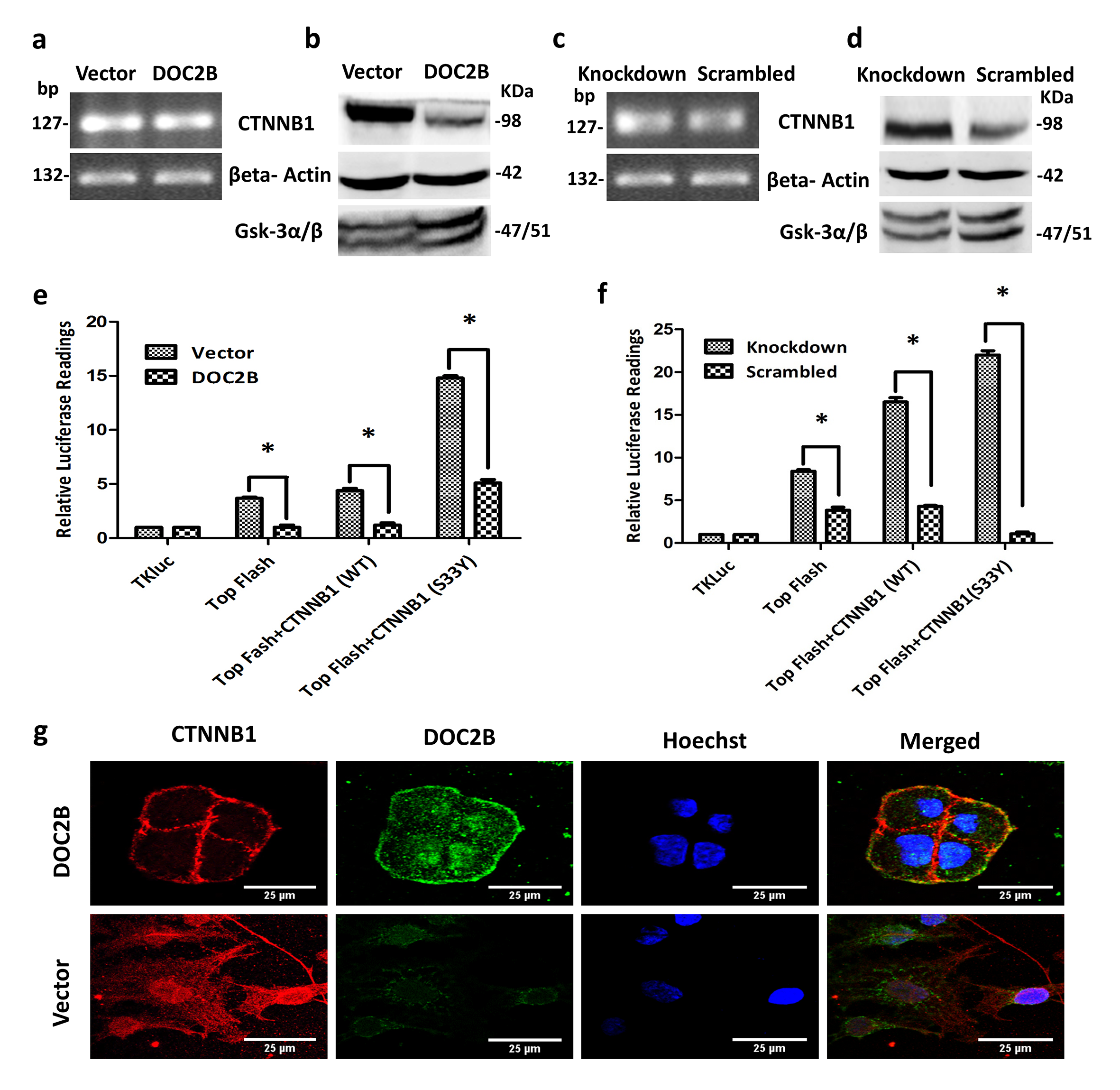

Supplement: Supplementary file 11 — DOC2B suppresses β-catenin signaling and β-catenin induced TCF/LEF activity. A and C) Relative expression levels of CTNNB1 in the DOC2B overexpressing and knockdown cells when compared to their respective controls as measured by semi-quantitative RT-PCR. B and D) Western blot analysis for CTNNB1 and GSK3A/B in DOC2B overexpression and knockdown cells respectively. E and F) Dual luciferase assay showing the effect of DOC2B on TOPFLASH / FOPFLASH activity in DOC2B overexpressing SiHa cells and DOC2B knockdown Cal27 cells respectively. TCF activity was repressed from 3.2 ± 0.32 to 1.04 ± 0.43 fold in the presence of DOC2B when compared to control SiHa cells (P = 0.02). In contrast, the knockdown of DOC2B enhanced the TCF activity from 3.72 ± 0.23 to 8.15 ± 0.44 fold (P = 0.006). Cells were co-transfected with TOPFLASH reporter and/or FOPFLASH reporter plasmid with mutant (S33Y) or wild-type (WT) CTNNB1 expression plasmid. The values were normalized to an internal Renilla luciferase. DOC2B significantly inhibits β-catenin transcriptional activity. In control cells, the TCF activity was found to be 4.2 ± 0.25 and 14.6 ± 0.58 fold in cells transfected with wild type and mutant β-catenin. In contrast, TCF activity was found to be 1.1 ± 0.386 and 4.8 ± 0.78 fold in DOC2B expressing SiHa cells transfected with wild type and mutant CTNNB1 respectively. The transfection of wild type and mutant CTNNB1 enhanced the TCF activity (16.1 ± 0.784 fold and 22 ± 0.41 fold) in cells transfected with wild type and mutant CTNNB1 in DOC2B knock down Cal27 cells as opposed to scrambled vector transfected Cal27 cells (3.86 ± 0.29 fold and 1.4 ± 0.19 fold in cells transfected with wild type and mutant CTNNB1) (P < 0.05). G) Representative confocal images of localization of DOC2B and CTNNB1 in control and DOC2B-overexpressing cells. CTNNB1 was co-localized along with DOC2B in the plasma membrane. DOC2B expression inhibited the nuclear translocation of CTNNB1. Results of each experiment are pre [file 10565_2021_9598_Fig14_ESM.png]

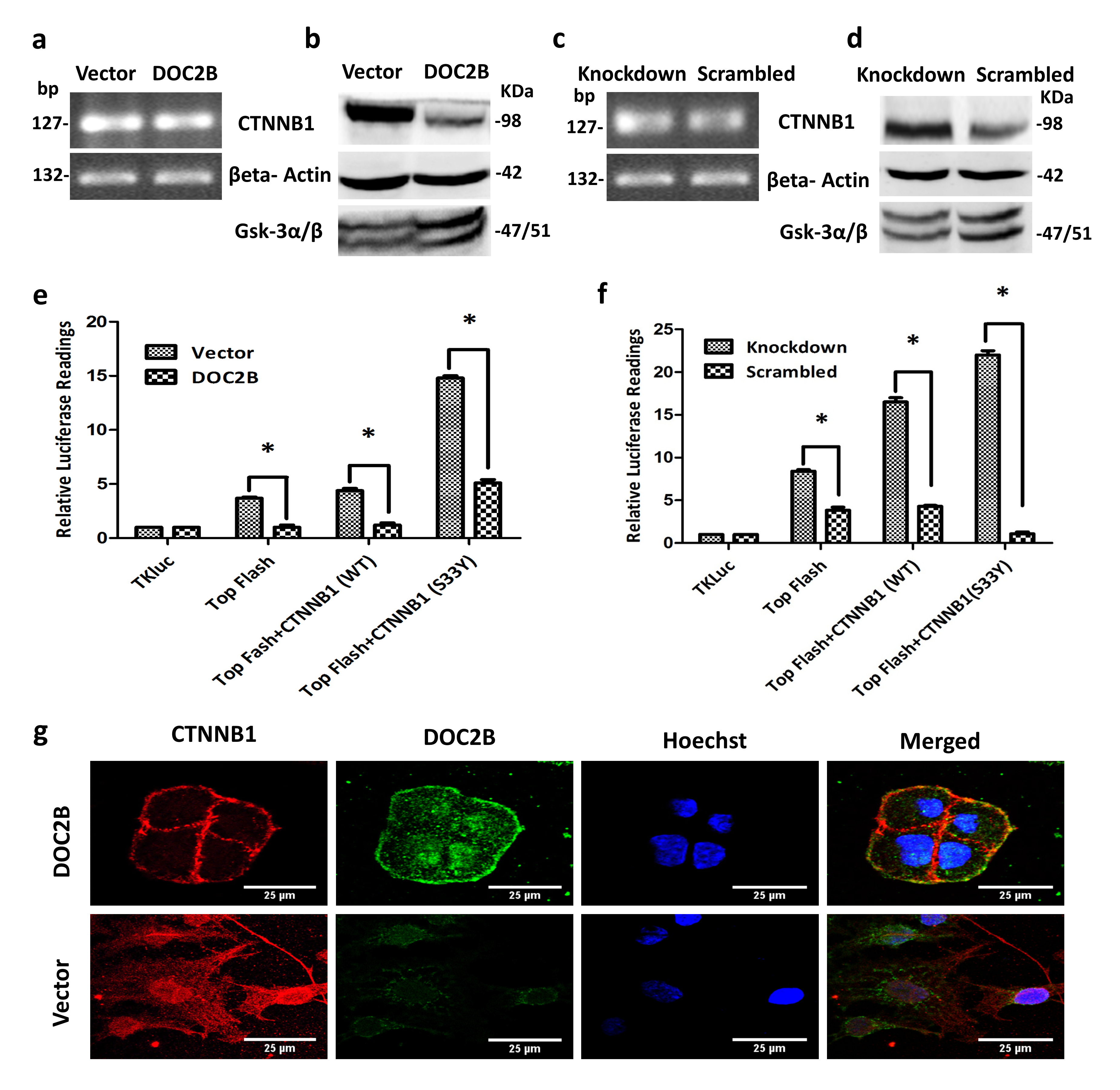

Supplement: Supplementary file 12 — High Resolution Image (TIF 4552 kb) [file 10565_2021_9598_MOESM6_ESM.tif]
